# Evidence for spreading seizure as a cause of theta-alpha activity electrographic pattern in stereo-EEG seizure recordings

**DOI:** 10.1371/journal.pcbi.1008731

**Published:** 2021-02-26

**Authors:** Viktor Sip, Julia Scholly, Maxime Guye, Fabrice Bartolomei, Viktor Jirsa

**Affiliations:** 1 Aix Marseille Univ, INSERM, INS, Inst Neurosci Syst, Marseille, France; 2 Assistance Publique - Hôpitaux de Marseille, Hôpital de la Timone, CEMEREM, Pôle d’Imagerie Médicale, CHU, Marseille, France; 3 Assistance Publique - Hôpitaux de Marseille, Hôpital de la Timone, Service de Neurophysiologie Clinique, CHU, Marseille, France; 4 Aix Marseille Univ, CNRS, CRMBM, Marseille, France; Newcastle University, UNITED KINGDOM

## Abstract

Intracranial electroencephalography is a standard tool in clinical evaluation of patients with focal epilepsy. Various early electrographic seizure patterns differing in frequency, amplitude, and waveform of the oscillations are observed. The pattern most common in the areas of seizure propagation is the so-called theta-alpha activity (TAA), whose defining features are oscillations in the *θ* − *α* range and gradually increasing amplitude. A deeper understanding of the mechanism underlying the generation of the TAA pattern is however lacking. In this work we evaluate the hypothesis that the TAA patterns are caused by seizures spreading across the cortex. To do so, we perform simulations of seizure dynamics on detailed patient-derived cortical surfaces using the spreading seizure model as well as reference models with one or two homogeneous sources. We then detect the occurrences of the TAA patterns both in the simulated stereo-electroencephalographic signals and in the signals of recorded epileptic seizures from a cohort of fifty patients, and we compare the features of the groups of detected TAA patterns to assess the plausibility of the different models. Our results show that spreading seizure hypothesis is qualitatively consistent with the evidence available in the seizure recordings, and it can explain the features of the detected TAA groups best among the examined models.

## Author summary

During evaluation, epileptic patients might be implanted with intracranial electrodes in order to gain insight into the initiation and evolution of the seizure. At the beginning of the seizure variety of electrographic patterns can appear, whose origins and significance is not yet fully understood, which makes the interpretation of the signals difficult. Here we look at one of the patterns, the so-called theta-alpha activity pattern, and propose that it can be explained by epileptic seizures gradually spreading across the cortex. To analyze this hypothesis, we compare three models of epileptic activity that can generate the theta-alpha activity pattern, and evaluate which of them is most consistent with the patterns found in the patients’ recordings. We simulate the seizure activity using the detailed geometry of individual patients’ brains and the location of the implanted electrodes, and we show that the spreading seizure model is most consistent with the observations. Such understanding can help the interpretation of the recordings in clinical practice, leading to better outcomes of resective surgeries.

## Introduction

### Intracranial EEG and early electrographic patterns

Intracranial electroencephalography (iEEG) is an essential tool in clinical evaluation of patients with focal drug-resistant epilepsy and its use in neuroscientific research is steadily growing [1, 2]. The objective of exploration using iEEG is to understand the spatiotemporal organization of the patient’s epilepsy with the goal to perform resective surgery and render the patient seizure free. In epilepsy both electrocorticography (ECoG) using the subdural grids and stereoelectroencephalography (SEEG) using the depth electrodes are widely employed.

The acquired intracranial recordings show variety of early electrographic seizure patterns, i.e. patterns occurring shortly after the appearance of electrographic seizure activity at the observed site. The patterns differ in frequency, amplitude, and waveform of the oscillations and in their temporal evolution. Several studies attempted to classify these patterns in an effort to distinguish between local onset and propagated seizures and to link the early patterns with different pathologies or with the outcome of a resective surgery [3–10].

### TAA electrographic pattern

**Fig 1:**
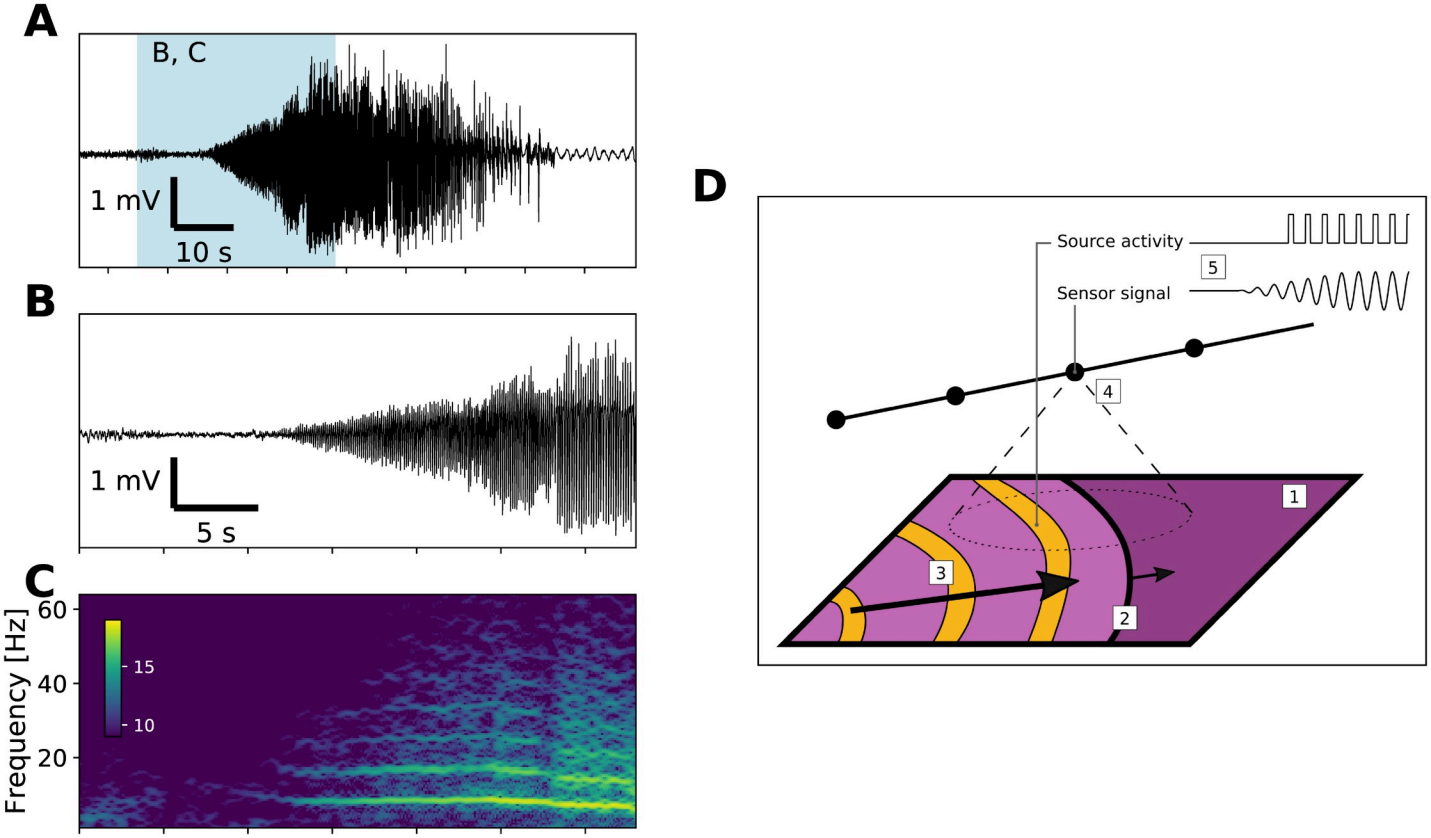
Example of the TAA pattern and the hypothesized mechanism. (A) Recording of a seizure from a contact of an implanted depth electrode in monopolar representation. Blue background marks the limits of the details in panels B and C. (B) Detail of the TAA pattern at the seizure onset. (C) Time-frequency representation of the signal in panel B (log transformed). The signal is dominated by oscillations around 8 Hz and its higher harmonics. (D) Hypothesized mechanism of the emergence of TAA pattern. During a seizure, cortical sheet (1) is gradually recruited into seizure activity via slowly progressing seizure wavefront (2). Inside the recruited area the abnormal activity is organized by fast traveling waves (3). Implanted sensors record the local field potential generated by nearby located cortical tissue (4), and through this spatial averaging, the rapid onset at the source level is transformed into gradual onset on the sensor level (5).

One of the often described patterns is what we call *theta-alpha activity* (TAA): an early ictal pattern characterized by sustained oscillations in the *θ* − *α* range with gradually increasing amplitude (Fig 1). Such pattern was reported under the names “rhythmic ictal transformation” [3], “sharp activity at ≤ 13 Hz” [6], and “theta/alpha sharp activity” [10]. Furthermore, several other studies include similar category of rhythmic theta-alpha activity, although without explicitly mentioning the gradually increasing amplitude [4, 7, 11]. In the seizure onset zone, the TAA pattern was reported as less common compared to low-voltage fast activity and low-frequency high-amplitude spikes patterns [3, 6, 10]. However, the pattern was commonly associated with the regions of seizure spread [4, 6, 7].

### Seizures spreading across the cortex

In this work we aim to investigate whether the TAA pattern can be produced by seizures locally spreading across the cortex. This local seizure spread, or propagating ictal wavefront, refers to the scenario where the seizure is initiated at a spatially restricted location (either autonomously or via external intervention), from where it gradually spreads to the surrounding tissue. Such seizure spread was observed in *in vitro* [12–14] and *in vivo* animal models of epilepsy [15], as well as in human patients [16, 17]. The reported velocity of the seizure spread was between 0.1 and 10 mm s^−1^, with the exception of situations where the lateral inhibition was suppressed at the time of seizure initiation. In that case the seizure spread velocity was reported to reach up to tens to hundred of mm s^−1^ [13, 14]; in this work, we do not model this special case. Theoretical models of epileptic seizure dynamics which reproduce locally spreading seizures exist, and include a network of coupled Wilson-Cowan units [18], spatially continuous version of the phenomenological Epileptor model [19], or a biophysically-constrained model of seizure dynamics [20]. The relevance of local propagation for large-scale spatiotemporal organization of seizures in human patients compared to the propagation through the long-range projections [21] was not yet determined.

Experimental results indicate that, at least under some conditions, the seizure activity in the recruited tissue has a distinct spatiotemporal organization formed by fast traveling waves of increased firing. Such waves were observed in the cortex of human patients during seizures, spatially extending not only across microelectrode arrays but also across ECoG array, with velocities ranging from 100 to 1000 mm s^−1^ [22–24]. Also this feature can be reproduced by some models of seizure dynamics [19, 20].

### Spreading seizures as a cause of TAA patterns

The two features of spreading seizures—slow wavefront and fast internal traveling waves—motivate our speculations that the locally spreading seizures may be linked to the observed TAA pattern, thus explaining its reported occurrence in the regions of seizure spread (Fig 1D). To better elucidate this possible link, one has to first consider the relation of the (unobserved) activity of the neuronal assemblies (to which we will hereafter refer as to the *source* activity) and of the signal recorded by the intracranial electrodes (hereafter referred as the *sensor* activity). The electrodes record the local field potential (LFP) generated by the neuronal assemblies both local and distant. The amplitude of LFP recorded on the sensors is affected both by the synchronization of the neuronal assemblies as well as by the geometry of the cortical tissue and subcortical structures and the exact positions of the contacts of the implanted electrodes [25, 26]. The LFP performs a spatial averaging of the source activity, and therefore the gradual recruitment of the neuronal tissue by the slowly propagating seizure may manifest itself as a gradually growing amplitude of the oscillations in the recorded signals—the characteristic feature of the TAA pattern. At the same time, if the internal organization of the seizure is dominated by the fast traveling waves, then the firing of the recruited tissue is synchronized by these waves. That, in turn, would give rise to LFP oscillations dominated by the single frequency, which is the second feature of the TAA pattern.

### Goal and organization of the paper

In this work we propose local seizure propagation as a (non-exclusive) candidate mechanism for the generation of electrographic TAA patterns. Here we aim to determine whether this hypothesis is plausible when confronted with the evidence available in the SEEG recordings of epileptic seizures in human patients. To do so, we take the following steps: First, we generate a synthetic data set of SEEG signals by simulating the seizure dynamics on realistic cortical surfaces using model parameters randomly sampled from prescribed parameter range. We use a simple model of spreading seizure as well as reference models of one and two homogeneous sources. Next, using a strict definition of the TAA pattern, we detect these in SEEG recordings obtained from a cohort of fifty subjects, as well as from the simulated SEEG. We then compare the statistical distributions of the features of the TAA patterns detected in the recordings with those detected in the simulated data set in order to assess the plausibility of the propagating seizure model relative to the reference models.

## Results

### Models of seizure activity

**Fig 2:**
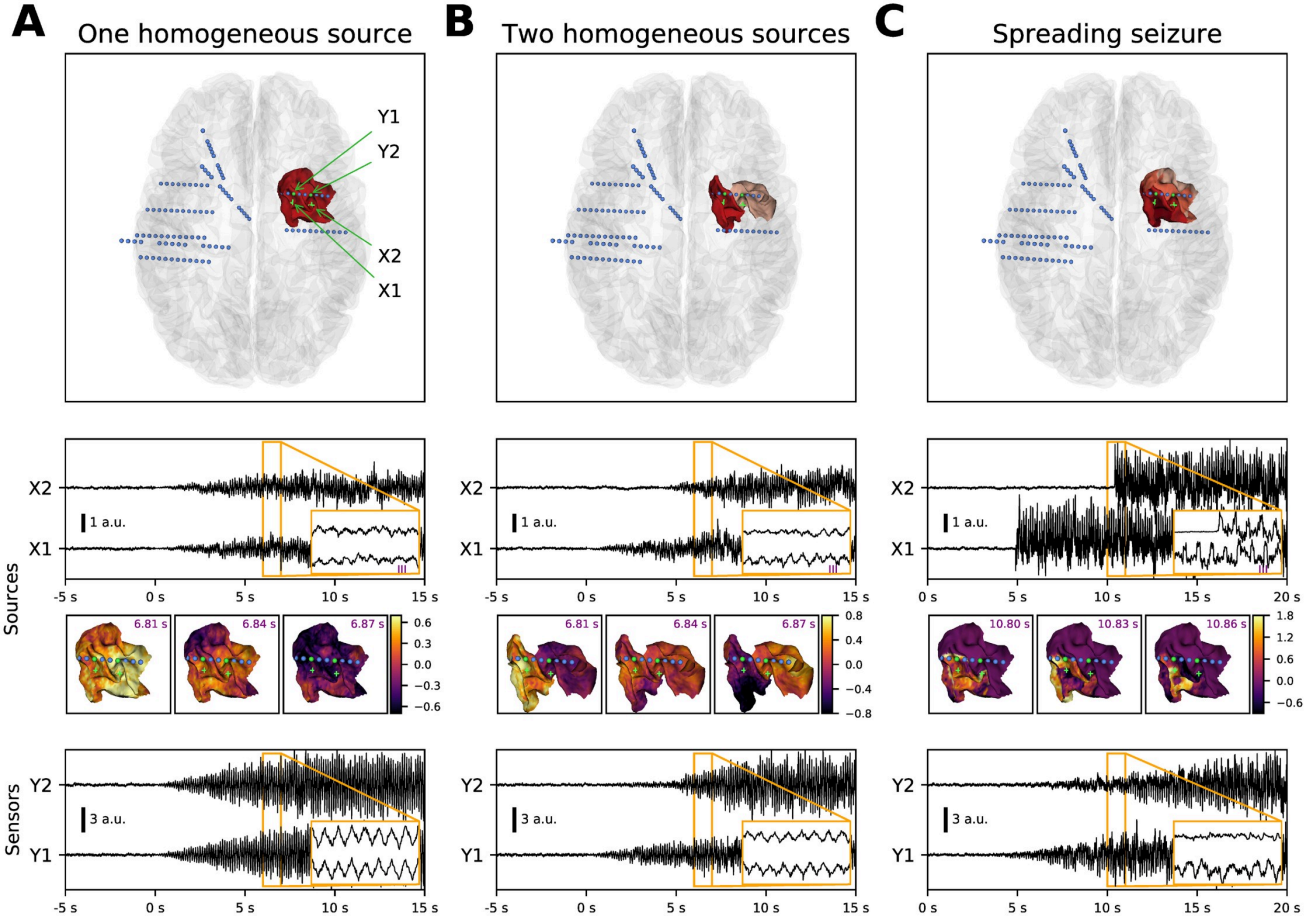
Models of the seizure activity. Each column presents an example of a simulated seizure with the different models in the noisy variation. From top to bottom, the panels show the position of the seizure patch and the implanted electrodes, source activity in two points on the patch, snapshots of the source activity (at time points marked in the inset above), and simulated sensor activity on two contacts located close to the seizure patch. (A) One homogeneous source model posits that the seizure activity is generated by one contiguous cortical patch, where any point follows the same dynamics (apart from the stochastic noise) with gradually increasing oscillations. (B) In the two homogeneous sources model, there are two patches recruited with a delay. On each patch any point follows the same dynamics with gradual onset as in the one source model. (C) In the spreading seizure model, the seizure activity start at a single point located on the cortical patch, and the seizure then slowly spreads until the whole patch is recruited (recruitment time is represented by the patch color in top panel). In every cortical unit represented by a vertex of the triangulation the seizure activity starts instantly with no transition period (second panel). Despite this rapid onset on the source level, the onset of the seizure activity at the sensor level is gradual (bottom panel). This is due to the spatial averaging effect of the measured local field potential, which transforms the slow spatial spread of the seizure into gradual onset in a sensor signal. The lowermost panel shows that all models produce SEEG signal resembling the TAA pattern.

For our analysis, we used three models of seizure activity—one homogeneous source, two homogeneous sources, and spreading seizure (Fig 2). All models follow the same structure. They posit that only a small patch (or two small patches in case of the two sources model) of the cortex is recruited in the seizure activity, and the rest of the cortex is modeled just as a noise source. The source activity is represented on the vertices of the triangulated surface, and differs for the three models. The *spreading seizure model* (Fig 2C) represents the main hypothesis that the patch is recruited gradually as the seizure spreads, as described in the introduction. On the source level, the seizure starts instantly with no transition period. Inside the recruited part of the patch, the activity is organized by fast traveling waves. With this model, the typical TAA feature—gradual increase of oscillations amplitude—is present only on the sensor level, and emerges due to gradual recruitment of the cortical tissues and the sensor to source projection.

As a comparison, we introduce two reference models. Both can reproduce the TAA pattern on a single sensor, however, the features of the TAA groups (that is multiple TAA patterns detected on one electrode) might differ. The first control case is the *one homogeneous source model* (Fig 2A). This model represents the alternate hypothesis that the characteristic TAA features—again, most notably the gradual increase of the amplitude—are already present on the source level. This model conceptually corresponds to the neural mass models in which the spatial component of the dynamics is absent and thus not crucial for understanding the phenomenon in question. For one homogeneous source model all vertices in the seizure patch follow the same dynamics of oscillations with gradually increasing amplitude.

The model can produce the TAA pattern, yet the properties of the generated TAA groups are restricted. For instance, due to all signals coming from a single source, the signal will be highly correlated, and large delays between the appearances of the TAA patterns cannot be expected, unlike in the spreading seizure model. Thus, to provide more compelling control case we also introduce a *two homogeneous sources model* (Fig 2B). In this model two cortical patches, each of them internally homogeneous, are recruited at different times. The patches oscillates with the same frequency, although possibly with different phases. Such model still represents a conceptual link to the neural mass modeling, except now with two neural masses. Again, the TAA features are already present on the source level, and the spatial component does not play major role in their emergence. Unlike to single source model, however, the model can be expected to produce TAA groups with delays between the TAA appearance, or less correlated signals.

Furthermore, the features of the TAA groups can be influenced by the level of noise. For instance, even the one source model can produce sensor signals with low correlations if, on the source level, the spatially homogeneous oscillations are superposed with spatially dependent noise. To assess this noise effect, we consider all models with noise-free and noisy seizure activity. For the latter, on top of the deterministic source activity spatially correlated pink noise is added.

The simulations were performed on the triangulated cortical surfaces obtained from the fifty subjects in the patient cohort. The triangulated surfaces had mean total area 1790.7 cm^2^ (standard deviation 204.6 cm^2^), mean number of vertices 251.9 thousands (s.d. 31.2 thousands), mean number of triangles 503.1 thousands (s.d. 62.4 thousands), mean triangle area 0.356 mm^2^ (s.d. 0.199 mm^2^), and mean edge length 0.924 mm (s.d. 0.295 mm). To assess the influence of the triangulation density, we performed a set of simulations on this standard triangulation and refined triangulation, obtained by splitting every existing triangle into four (S1 Fig). The results show that using the standard triangulation does not introduce differences of higher order of magnitude than those due to the stochastic background noise, and we thus used the standard triangulation in the rest of the work.

For each subject and each model, 300 simulations were performed, leading to a total of 15000 simulated seizures for each model. For each simulation, the model parameters were drawn randomly from the prescribed parameter range (see Methods). The source activity was projected on the sensors using the dipole model of generated local field position. The position of the contacts was derived from patient data and thus constituted a realistic placement of the electrodes. In case that the seizure activity was not detected on any contact in the simulation, new set of parameters was drawn and the simulation was repeated.

### Detection of TAA patterns

**Fig 3:**
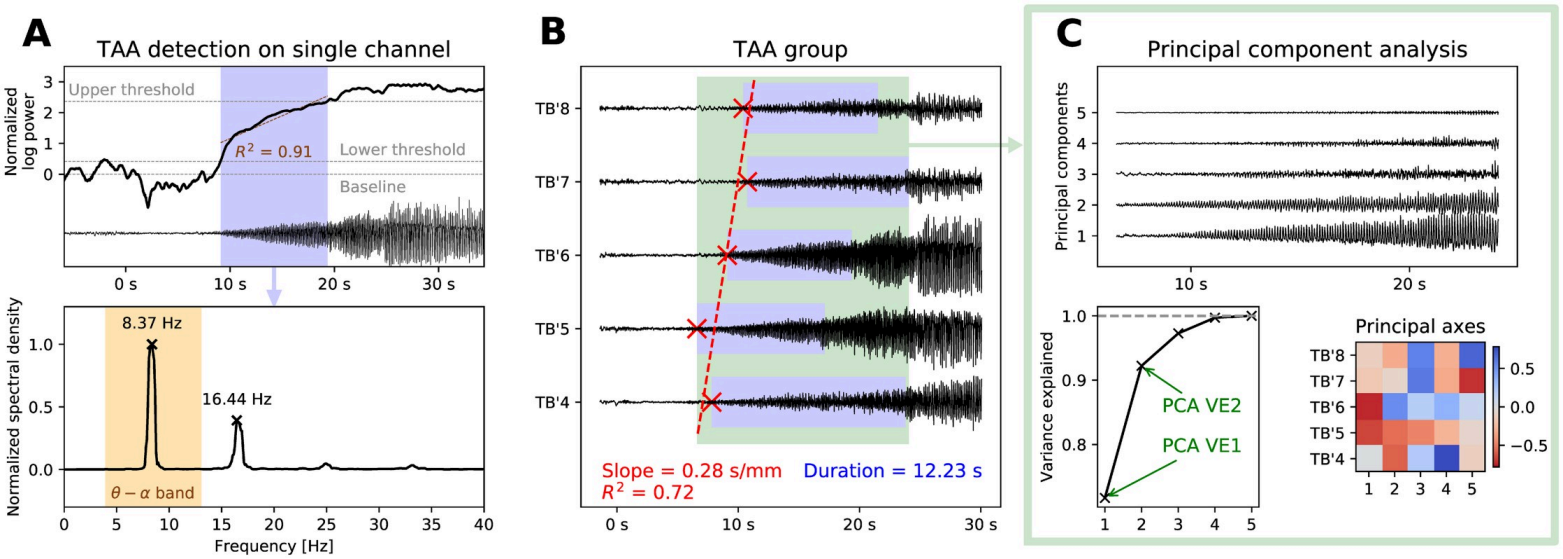
Detection of TAAs and extracting the features of a TAA group. (A) For each channel, we calculate the normalized log-power in the *θ* − *α* band and determine the period of growth (in blue) from the baseline to full seizure activity. We then test if the growth is roughly close to linear (*R*^2^ > 0.75). If so, we check whether the largest peak of the normalized spectral density lies in the *θ* − *α* band and whether all other peaks are its harmonics. (B) When the TAA pattern is detected on four or more consecutive contacts on a single electrode, three features are extracted: the slope and coefficient of determination of the TAA onsets (in red) and average duration of the TAA patterns (in blue). The figure shows an example on five contacts of the electrode TB’ implanted in the left temporo-basal cortex. (C) In the TAA interval, the principal component analysis of the SEEG signals is done to extract two more features: the variance explained by the first one and first two components.

Using a cohort of 50 patients with focal epilepsy who underwent clinical evaluation via SEEG, we detected the occurrences of the TAA pattern in the seizure recordings as well as in the simulated SEEG signals (Fig 3A). These patterns were detected on all channels separately. Next, we restricted our analysis to the TAA patterns occurring on at least four consecutive contacts on a single electrode, which we in the following text call *TAA groups*. This step was motivated by the reasoning that the source activity often affects multiple electrode contacts at once, and that more information about the spatial configuration of the sources can be extracted from the properties of these groups. Strictly speaking, this restricts our subsequent conclusions only to TAA instances occurring in groups. Panel A in S2 Fig however shows that the majority of TAA instances do not occur in isolation even if they do not pass our criteria for group occurrence. Furthermore, analysis of the frequencies, durations, and delays from the seizure onset of the group and non-group TAA instances does not indicate a difference between these two populations (S2 Fig, panel B), giving basis for the belief that the conclusions can be extended to all TAA patterns.

From these TAA groups we extracted five features on which our analysis is based: slope of the onset of the TAA patterns and the coefficient of determination as obtained by linear regression, average duration of the TAA patterns, and the variance explained by first two PCA components (Fig 3B and 3C). The choice of these features was motivated by the goal of assessing the plausibility of the spreading seizure model. The first three features (slope, *R*^2^, and TAA duration) are informative about the hypothesized seizure spread (or its absence): High values of slope indicate slow unidirectional linear spread, while low values are indicative of either near simultaneous onset, or nonlinear spread. The coefficient *R*^2^ quantify how linear the spread is. We note, in particular, that a bidirectional spread from the group center would be described by zero slope and low *R*^2^, and thus not well captured by the selected features. We have however avoided adding features characterizing such nonlinear spread, since we deemed the possible features not sufficiently robust when computed from the noisy TAA onsets in groups containing as few as four contacts. The duration reflects the rate of spread; in general, slowly spreading seizure would result in larger TAA duration, however this is also affected by the geometrical configuration of the cortical surface and the relative position of the electrode contacts. The results of the principal component analysis are informative of the internal organization of the seizure activity. For example, high values of variance explained by the first component could indicate that the signals are generated by a single point source, or an extended synchronized source. Low values, on the other hand, could be indicative of multiple sources, or structured but inhomogeneous activity (such as the fast traveling waves). Similarly for the variance explained by the second component. Again, the values are influenced not only by the source activity, but also the geometrical configuration of the sources and sensors.

**Fig 4:**
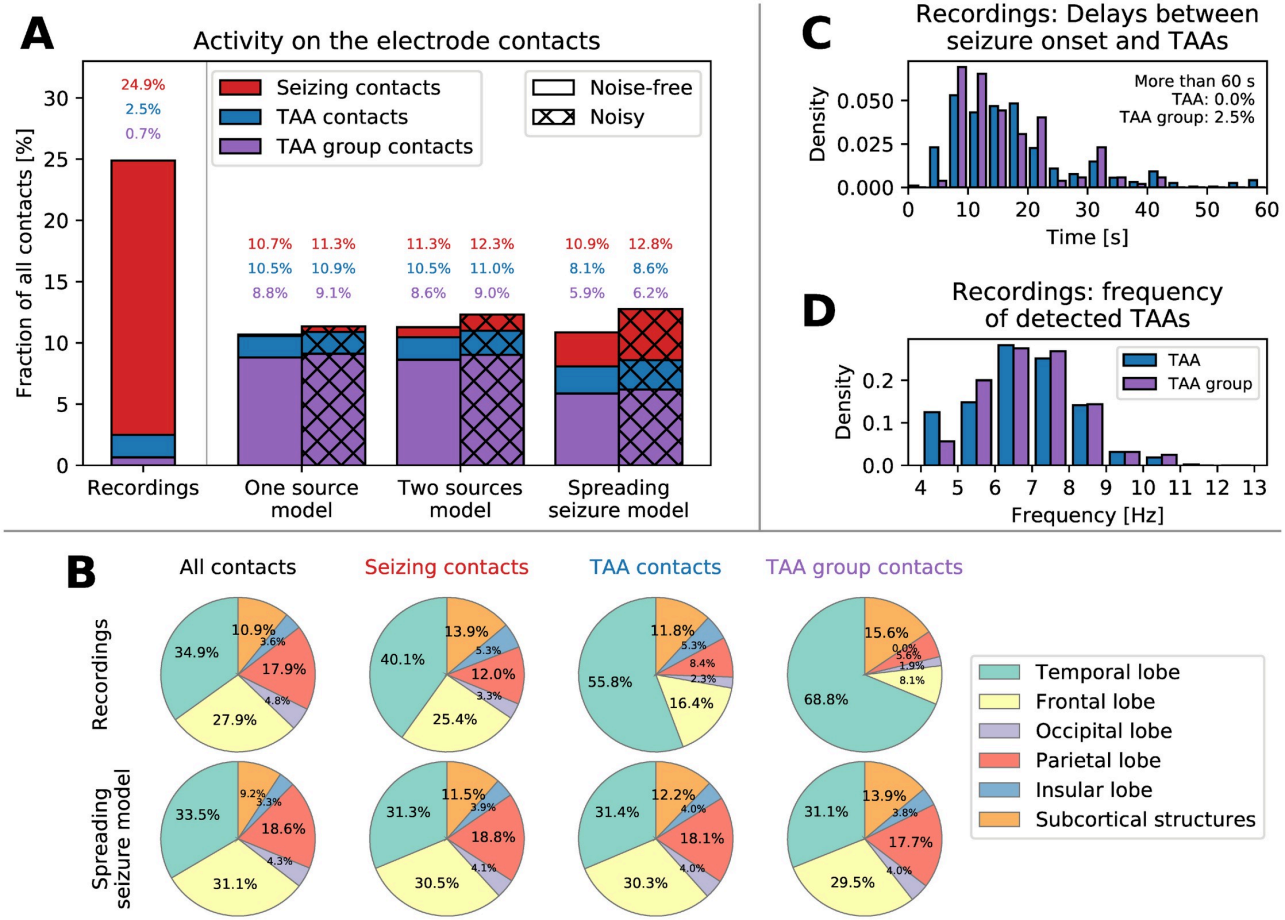
(A) Classification of the contacts based on the recorded/simulated activity. Seizure activity was detected on around 25% of contacts in the recordings and around 10% in the simulations. The TAA pattern was detected on a subset of these seizing contact, and contacts that belong to a TAA group formed even smaller subset. (B) Location of the contacts in the brain. In the spreading seizure model, the TAA patterns are distributed homogeneously in the brain, following the implantation (first column). In the recordings, the TAA patterns occur dominantly in the temporal lobe. (C) Delays of the TAA pattern onset relative to the clinically marked seizure onset in the recordings. Majority of the TAAs appear between eight to twenty second after the seizure onset. (D) Frequencies of the oscillations in the TAA patterns detected in the patient recordings.

Fig 4 summarizes where and when the TAA patterns were detected, and Table 1 summarizes the number of detected TAA groups for the models and the recordings. In the recordings, the TAA pattern was detected on around 2.5% of channels (around 10% of channels where seizure activity was detected), only around one quarter of these were in a TAA group (i.e. more than four TAA patterns on the consecutive contacts of a single electrode). In contrast to that, in all computational models the TAA pattern was detected on majority of contacts with seizure activity (Fig 4A). That is however to be expected from the models designed to produce the TAA patterns.

**Table 1:** Number of detected TAA groups in the simulated and recorded SEEG. Number in the bracket is the number of simulated or recorded seizures, in which these patterns were searched for.

Model	Noise-free simulations	Noisy simulations
One source	30023 (15000)	31002 (15000)
Two sources	29268 (15000)	30589 (15000)
Spreading seizure	22426 (15000)	23578 (15000)
Recordings	32 (204)	

Analysis of the location of the contacts where the TAA patterns were detected reveal that they dominantly occur in the temporal lobe (Fig 4B). This observation cannot be explained by a biased electrode implantation—the ratio of the number of contacts where TAA pattern was detected and the number of implanted contacts is higher in the temporal lobe. That is something that the spreading seizure model cannot reproduce. Most of the TAA patterns appear more than eight seconds after the clinically marked seizure onset (Fig 4C), supporting the hypothesis that TAA pattern is a propagation pattern. The frequency of the oscillations during the TAA patterns is restricted mainly to the interval 5 to 9 Hz (Fig 4D). The analysis of the location of TAA patterns in relation to the suspected epileptogenic zone identified by the Epileptogenicity index [27] does not reveal any substantial differences from the location of the seizing channels without the TAA pattern (S3 Fig).

### Features of the recorded and simulated TAA groups

As demonstrated on Fig 2 (bottom panels), all models can produce activity which at least visually resembles the TAA pattern. To quantify how well the models fit the empirical data, we detected the TAA patterns in the simulated SEEG in the same way as in the patient recordings, and, for each of the detected TAA groups we extracted the five group features (Fig 3B and 3C). This procedure gave us for each detected TAA group (in recordings as well as all models) a five-dimensional feature vector, or TAA group “fingerprint”. On S4 Fig we analyze the similarity and variability of the TAA groups detected in the recordings using clustering analysis, showing that while differences between the detected TAA groups exists, no single subject exhibits TAA groups that would be unique in their features and could be consider an outlier.

**Fig 5:**
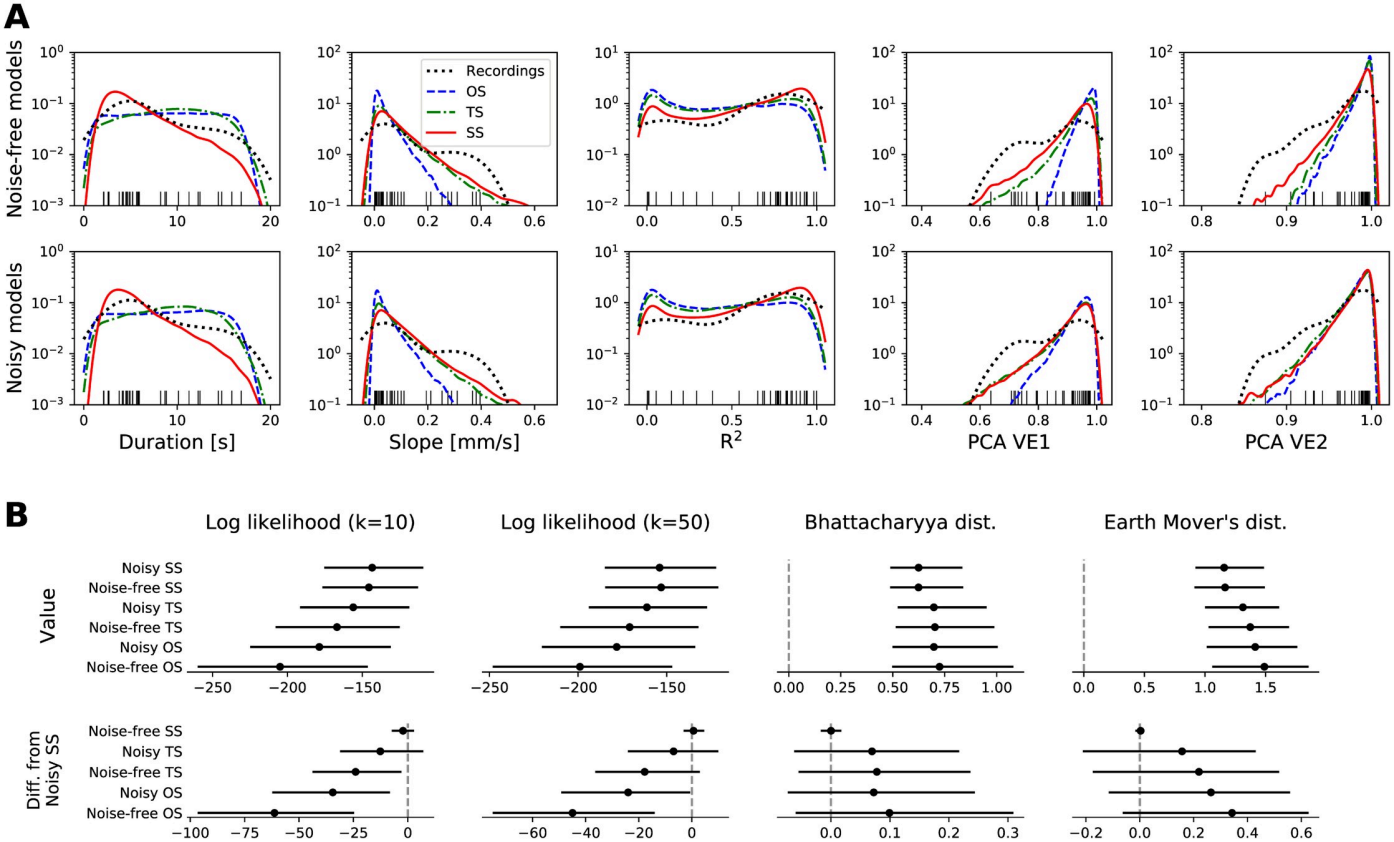
(A) Features of the detected TAA groups in the recordings and simulations. Each panel shows the density of one of the five features of the TAA group (see Fig 3) obtained from the samples via kernel density estimation for noise-free (top row) and noisy (bottom row) simulations. Each black tick at the bottom edge corresponds to a TAA group in the recordings; these ticks match the density marked by the black dotted line, and are the same in both rows. (B) Quantification of the fit of the models to the empirical data. The four columns show the median and the confidence intervals at 95% level of four measures of a goodness-of-fit: k-nearest-neighbor estimation of log-likelihood (with *k* = 10 and *k* = 50; higher is better) and Bhattacharyya and Earth Mover’s distances (lower is better). Upper panels show the values itself, lower panels show the difference to the noisy spreading seizure model. To calculate the Bhattacharyya and Earth Mover’s distances, the samples were binned into 1024 uniform bins (each dimension divided into four bins) with limits indicated by the outermost ticks on x-axes in panel A. The confidence intervals are obtained by bootstrapping, that is repeatedly calculating given measure with a random resample with replacement from the original 32-element sample of detected TAA instances. Number of resamples was 1000, except for the Earth Mover’s distance where only 100 was used due to the computational demands. Abbreviation of the model names: OS—One homogeneous source, TS—Two homogeneous sources, SS—Spreading seizure.

Fig 5A shows the densities of the five group features as observed in the recordings and in the simulations. We highlight several points of interest:

- Short TAAs are more numerous (panel Duration). The empirical distribution of the TAA duration is skewed towards shorter TAAs with duration of 3-7 seconds, and this aspect is reproduced by the spreading seizure model. In contrast, the duration of TAAs in the one and two source model is determined by the prescribed uniform distribution.
- Sequential recruitment is rare even under spreading seizure model (panel Slope). The delayed appearance of the TAA pattern on neighboring contacts would be the most predictive sign of a spreading seizure. Yet, as our model demonstrates, it is a relatively rare event due to the spatial constraint imposed upon the alignment of the electrode with the direction of seizure spread.
- Majority of TAA groups are highly correlated (panel PCA VE1). The trend is reproduced by the two source model as well as the spreading seizure model, although they both predict higher number of highly correlated groups, even in their noisy variations.

### Empirical data are most consistent with the spreading seizure model

Given the “fingerprints” of the TAA groups (i.e. the samples from the distributions on 5-dimensional space of TAA group features) obtained from the recordings on one hand and from the simulations on the other hand, we want to quantify the distance between the recording distribution and all model distributions. Both the recording distribution and the three model distributions are known only via finite amount of samples from them (Table 1). The number of samples in the empirical distribution (*n* = 32) is determined by what was detected in the data, while the number of samples in the model distributions depends on the number of simulations performed; this we set considering the practical limits of computational resources.

Since any single measure of goodness-of-fit has its own advantages and disadvantages, we employ four different measures to assure that the results are robust: *k*-nearest-neighbor approximation of the log-likelihood (with *k* = 10 as well as *k* = 50), Bhattacharyya distance and Earth Mover’s distance (Fig 5B). The log-likelihood measures how probable are the empirical samples given the model. The Bhattacharyya distance measures the overlap of two probability distributions. For two non-overlapping distributions it gives infinite value, unlike the Earth Mover’s distance, which takes into account how far apart the probability masses are. To quantify the uncertainty in the distance estimate, we perform the bootstrapping, that is, we repeatedly calculate the measure for a random resample of the empirical samples.

We point out that the data sets are paired, that is, the same resamplings of the empirical observations are evaluated under the different models. The unpaired comparison of goodness-of-fit measures (Fig 5B, upper panels) does not take this into account, and while it shows differences in medians indicating best fit of the spreading seizure model, the overlapping confidence intervals do not demonstrate statistical significance. With the pairwise comparison we control for the variance between the different resamplings, which leads to higher statistical power. Here we compare with the most plausible noisy spreading seizure model (Fig 5B, lower panels), and this pairwise comparison reveals statistically significant differences on the chosen confidence level of 95%, however, only for the log-likelihood measures. The inconclusiveness of the results for Bhattacharyaa and Earth Mover’s distance can be in part assigned to the coarse binning used for their calculation, which is however necessary due to the computational demands. The log-likelihood measures prefers the spreading seizure model more strongly, however, the noisy two source model cannot be ruled out at the chosen confidence level. The results thus call for analysis on a larger data set to obtain higher statistical power. We note that qualitatively consistent results are obtained also with modified parameters of the TAA detection procedure (S5 Fig), illustrating the stability of the results.

### Analysis of the spreading seizure model

**Fig 6:**
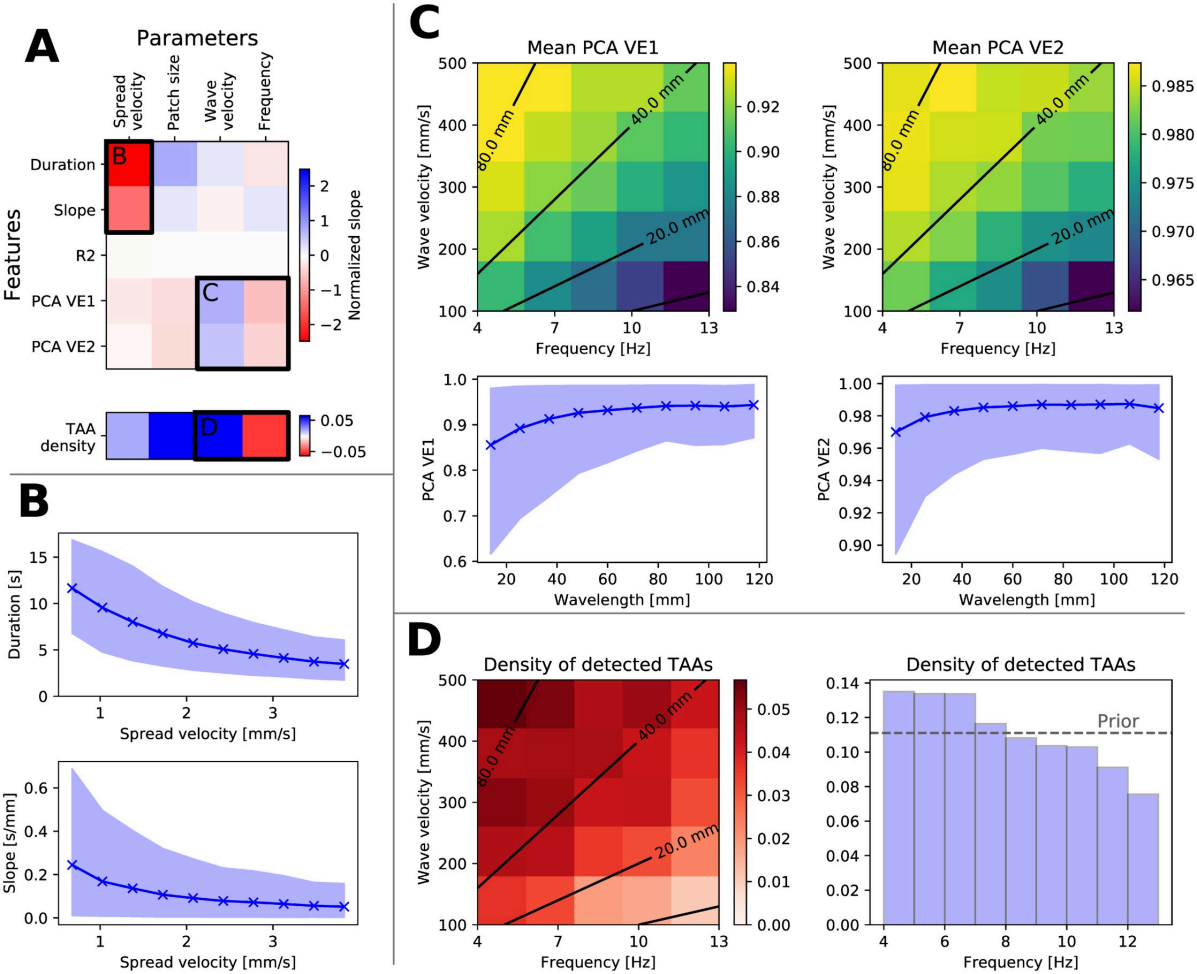
Effects of the model parameters on the features of detected TAAs in the spreading seizure model in the noisy variant. (A) Upper plot shows the slope of linear regression *s*_*p*,*f*_ between all parameters and features, normalized by the parameter range width *w*_*p*_ and standard deviation *σ*_*f*_ of the feature *s*_*p*,*f*_ *w*_*p*_/*σ*_*f*_. Lower plot shows the normalized change in parameter mean for detected TAAs and the prior distribution, $(\mu_{p}^{TAA}-\mu_{p}^{prior})/w_{p}$, indicating shift of the parameter density to higher/lower values. More saturated blue (red) elements indicate stronger positive (negative) relation. Labeled regions are analyzed in other panels. (B) Relation between the spread velocity and duration of the TAAs and slope of the TAA spread. Solid line and points represent the mean of the features, the shaded area is the 10-90 percentile range. (C) Relation between the frequency and wave velocity and variance explained by the first one and first two components. In the upper plots, black contours indicate the wavelength = wave velocity / frequency. The relation between the features and the wavelength is shown in the lower panels. (D) Relation between the frequency and wave velocity and the density of detected TAAs. The number of detected TAAs decreases with shorter wavelength (left panel) as well as with the frequency alone (right panel).

For the spreading seizure model, indicated as most plausible explanation of the data, we investigated the cause-effect relation between the model parameters and the features of the model-generated TAA instances. Such analysis can be valuable for future studies; if, for example, one attempts to perform a model inversion for any individual TAA instance, detailed understanding of the model behavior will be critical for success. In order to evaluate these cause-effect relations, we performed linear regression between the model parameters and model-generated features. Fig 6A shows the results, while rest of Fig 6 analyzes the important relations revealed, and S6 Fig shows the relations between all parameters and features.

The strongest relation indicated is between the spread velocity and the TAA duration and the slope of the TAA spread (Fig 6B). This can be understood by considering that faster spreading seizures recruit the whole patch in shorter time, leading to a shorter duration of the TAA pattern, as well as to lower slope of the TAA onsets on neighboring contacts. The second parameter, patch size, has positive effect on the TAA duration since the patch recruitment requires longer time, and negative effect on the variance explained by the first and second PCA component. That can be interpreted that smaller patches behave more as point sources, generating highly correlated signals on all electrodes which has high values of variance explained.

A further indicated strong relation is between the wave velocity and the frequency of the oscillations on one side and the variance explained by the PCA components on the other side (Fig 6C). The role of the two parameters is best understood when considered together, as they define the wavelength of the fast waves, *λ* = *u*_*wave*_/*f*. In the simulations with longer wavelengths, larger areas of the seizing patch are synchronized together, that is, they oscillate with similar phase. The generated local field potential around the seizing patch is dominated by these common oscillations, leading the high variance explained by the first PCA components, and vice versa for the shorter wavelengths.

### Higher frequency TAAs are less observed in the spreading seizure model

The spreading seizure model can explain a phenomenon observed in the intracranial recordings, which is that, for frequencies above 7 Hz, the TAA patterns with higher frequencies appear less often then those with lower frequencies (Fig 4D). In particular, only a handful of TAA instances with frequencies above 10 Hz. This phenomenon, although to a lesser degree, can be seen also in the TAA instances generated by the spreading seizure model (Fig 6D, right panel), despite the frequency in the simulations being sampled uniformly from 4 to 13 Hz range.

In the simulations, this observed effect can be explained again by considering the wavelength of the fast waves. With shorter wavelengths (and higher frequencies), the patch oscillates with different phases, and in the generated local field potential these oscillations effectively cancel each other. In effect, the high frequency oscillations are less visible and less often detected. These results indicate a possibility that, whatever is the mechanism of the underlying source oscillations causing the TAA pattern, it might not be restricted to the frequency range 4 to 13 Hz commonly associated with the TAA pattern. Rather it might be that the higher frequency oscillation do occur on the source level, yet they go undetected at the sensor level due to the spatial canceling. Such argument however only applies for the upper limit of the frequency range, as nothing precludes the detection of the lower frequency oscillations.

## Discussion

### Summary of the results

In this work we examined the hypothesis that the TAA pattern is caused by seizures spreading across the cortex. The hypothesis ties together the observed SEEG pattern with the observations of the spreading seizures [16, 17] and fast traveling waves during seizures [23, 24] in fashion consistent with recent models of seizure dynamics [19, 20]. We analyzed the SEEG recordings of epileptic seizures collected from a cohort of fifty patients and compared how the predictions of three different models fit the data. Among the tested models, the predictions of the spreading seizure model in the noisy variation were closest to the observed data. The model with realistic parameters is consistent with most features of the observed TAA patterns, including a) the duration of the TAA patterns of several seconds (Fig 5A, panel Duration), b) rarely observed sequential recruitment of neighboring contacts (Fig 5A, panel Slope), or c) the high percentage of TAAs with highly correlated signals (Fig 5A, panel PCA VE1). In addition, the model may provide an explanation for the upper limit of the TAA frequency range (Fig 6D). There are however several observations that the spreading seizure model cannot explain, mainly why are the TAA patterns disproportionately often observed in the temporal lobe (Fig 4B).

### Relation of TAA and spreading seizures

Our results show that the spreading seizure model is the most plausible explanation when looking at all detected TAA patterns as a group. That, however, does not mean that it is the best explanation for any single TAA even among the limited selection of models we have evaluated. One thus cannot conclude that all TAA occurrences are caused by spreading seizures; other explanations might be plausible for any individual case. Indeed, TAA patterns were observed not only in the sites of seizure propagation, but also within the epileptogenic lesions [10], where the explanation by spreading seizure might be questioned.

One might consider fitting the models for individual TAA patterns using the information about the geometry of the cortex in the vicinity of the electrode of interest in order to obtain the parameter values best explaining the individual pattern. These parameters might include, in case of the spreading seizure model, the velocity and direction of the spread or the location of the seizure patch. The fitted models could then be compared to establish the probable cause of each pattern. This effort would however be complicated by factors shared by many inverse problems, mainly large degree of freedom, ill-posedness and issues linked to identifiability [28, 29]. To deal with such issues, strong regularization of the model corresponding to strong assumptions about the nature of the source activity would probably be needed, limiting the usefulness of the results.

### Frequency of occurrence of TAA patterns

In the seizure recordings the TAA pattern was detected in 10% of the channels with seizure activity. That is partly a consequence of the utilized detection mechanism: on purpose we have used relatively strict criteria to define what is a TAA pattern in order to get TAA patterns from which the features can be robustly extracted. If, instead, we opted to classify all channels according to the electrographic patterns into several distinct categories, more questionable patterns could have been classified as TAA patterns as well and the percentage could be higher. Indeed, Schiller and colleagues [4] found that in the sites of seizure propagation in mesiotemporal lobe, 53% of early patterns consisted of “rhythmic round theta and delta activity” correlated with activity at the onset site, possibly related to the TAA pattern as we defined it here. In the sites of seizure propagation in neocortex the proportion was even higher (82%). Also Perucca and colleagues [6] state that the TAA pattern was found in the propagation sites in more than half of the seizures.

### Thalamic involvement

In this work we do not explore the biological or dynamical mechanisms of the traveling waves, only its manifestations on the intracranial recordings. While the traveling waves might be generated and sustained purely by the cortex as predicted by some models of seizure dynamics [19, 20], they might be also driven by or arise from interactions with other structures. One such structure could be the thalamus. Thalamus was for long implicated in the generation of rhythmic spike-and-wave discharges in absence seizures [30, 31] but evidence exists for its involvement in focal seizures as well [32–34]. Interestingly, experimental and computational evidence show that thalamus can support propagating waves, both in isolation or as a part of a coupled thalamocortical system [35]. Its involvement in temporal lobe epilepsies [32] could explain the predominant occurrence of the detected TAA patterns in the temporal lobe.

### Modeling of TAA patterns in large scale brain models

Although the forward problem, aka map between source and sensor space, is clearly acknowledged as a contributor to the difficulty in performing correctly model inversion, often such recognition remains academic. Clinically, SEEG electrode contacts are regularly identified synonymously with brain region locations (electrode contacts A1-A2 for right hippocampus, B1-B2 for right amygdala, etc.), although there is a well-established variability in terms of surgical implantation and subsequent clinical interpretation, with consequences for surgery success rate and scientific reproducibility, in case the data are used for research. Our contribution highlights the importance of understanding the intracranial signals (and TAA patterns specifically) as signals generated by spatiotemporal dynamics, that is, by activity supported on spatially extended cortical tissue and exhibiting spatiotemporal phenomena such as wavefronts or waves with specific direction and velocity. Present results open the door for further efforts, such as precise localization of the involved cortical tissue and the wavefront direction and velocity for any TAA instance, utilizing the patient-specific geometry of the cortical folding. Success in this effort would lead to better understanding of the pathways of seizure propagation in individual patients, and possibly improved localization of the epileptogenic zone.

Epilepsy research is particularly deeply connected to nonlinear dynamics. One of the few fundamental discriminants of nonlinear dynamics is sudden qualitative changes in behavior, which are technically described by bifurcations [36–38]. They are powerful tools of modeling, because the behavior of any dynamic system can be smoothly mapped upon a set of canonical equations (so-called normal form) that is representative for the transition. In epilepsy, there is large evidence that the majority of seizure onset and offset transitions can be understood as bifurcations [39–41], although also other forms of transitions have been recognized, for instance in absence seizures so-called false bifurcations, in which significant changes occur rather smoothly than discretely [42]. Bifurcation analysis permits the construction of a seizure taxonomy based on purely dynamic features [40, 43], which guides further research and may, for instance, lead to the discovery of novel attractors [44]. From the perspective of bifurcations, the TAA patterns would be classified as a dynamical system undergoing a supercritical Hopf bifurcation, whose distinguishing feature is the gradually increasing amplitude of the oscillations after crossing the bifurcation [40, 43]. As we have demonstrated here, the TAA pattern can also be produced by spreading seizures with fast traveling waves. It was shown that these features can be generated using the spatially extended Epileptor model [19], which uses the saddle-node bifurcation to initiate the seizure. This misidentification has two consequences: first, the extent of the oscillation source in a spreading seizure model changes over time, thus a simple spatial inversion assuming a static source is not appropriate and could lead to a misidentification of the epileptogenic zone; second, the bifurcation type may be incorrectly inferred, which has important consequences for the behavior of the dynamical system, such as the response to external stimulation, responses to local interventions via drug administrations and difference in network propagation. For instance, dynamical system theory predicts that stimulation of a system close to a supercritical Hopf bifurcation will not be able to trigger a seizure, whereas stimulation close to a saddle-node bifurcation generally will [38]. Such is a consequence of the different properties of multistability following from each bifurcation. Relevance of these theoretical observations for clinical practice is clear considering that electrical stimulation is used both for seizure suppression [45] and seizure induction aiding the epileptogenic zone localization [46]. Yet, because of the insufficient understanding of the underlying dynamical mechanisms, accurate prediction of the stimulation effects or their interpretation poses a limitation for more widespread application. This understanding remains restricted despite the promise of recent studies systematically linking the bifurcation theory with the in-vivo seizure dynamics [47] and with the response to electrical stimulation [48], and so the integration of spatial and spatiotemporal consequences of realistic forward modeling is an important element to be integrated in the future workflows.

### Limitations of the study

The results have to be interpreted within the limitations of the study. Importantly, it is the sample size. Even with a large cohort of fifty patients and multiple seizure recordings for each patient, only 32 TAA groups were detected in the recordings, and the model comparison was based on these 32 samples. Although it is only the model comparison that is affected by the small sample size and the model predictions are independent, the conclusions need to be considered in light thereof.

Further limitation is that the results of the model comparison inevitably depend on exact form of the models and the choice of the ranges of the model parameters. We have not attempted to fit the parameters to the observed data, instead, we chose the parameters distributions based on the experimental observations in the literature, and our choices could be questioned. We therefore advise not too interpret the results in an overly formal way such as evaluating Bayes factors leading to the statements on the strength of evidence in favor of one or the other model [49]. The main goal of the work was not to select the best model from the candidate models; such task is not even necessary. Rather it was to gain insight about the behavior, predictions, and shortcomings of the spreading seizure model when compared to the empirical data. The other models served us mainly as a baseline in this endeavor.

## Methods

### Ethics statement

The approval was granted by the local ethics comittee (Comité de Protection des Personnes Sud-Méditerranée I); the patients signed a written informed consent form according to its rules.

### Patient data

In this study we have used imaging and electrographic data from a cohort of 50 patients who underwent a clinical presurgical evaluation (S1 Table). The clinical evaluation was described in detail before [21]. The T1-weighted images (MPRAGE sequence, repetition time = 1900 ms, echo time = 2.19 ms, 1.0 × 1.0 × 1.0 mm, 208 slices) were obtained on a Siemens Magnetom Verio 3T MR-scanner. The patients were implanted with multiple stereotactic EEG electrodes. Each electrode has up to 18 contacts (2 mm long and 0.8 mm in diameter), which are either uniformly placed on the electrode, separated by 1.5 mm from each other, or placed in groups of five, which are separated by 9 mm. The location and number of electrodes varied between the patients depending on individual clinical considerations (mean number of electrodes 12.00, s.d. 2.65; mean number of contacts 125.92, s.d. 32.91). The SEEG was recorded by a 128 channel Deltamed system using at least 256 Hz sampling rate. The recordings were band-pass filtered between 0.16 and 97 Hz by a hardware filter. After the electrode implantation, a CT scan of the patient’s brain was acquired to obtain the location of the implanted electrodes.

### Cortical surface reconstruction

The brain anatomy was reconstructed from the T1-weighted images by FreeSurfer v6.0.0 [50] using the *recon-all* procedure. Afterwards, the triangulated surface used in this study was obtained by taking the midsurface of the pial surface and white matter-gray matter interface, i.e. the surface lying halfway between these surfaces. The position of the contacts in the CT scan with implanted electrodes was marked using the GARDEL software [51], and then transformed to the T1 space using the linear transformation obtained using the linear registration tool FLIRT from the FSL toolbox [52].

### Computational models

The models define the source activity on the subject’s cortex Ω, and its projection on the sensors implanted in the brain. Specifically, the models prescribe the seizure activity on the recruited part Ω_*r*_(*t*) of the excitable patch Ω_*e*_ of the cortex, Ω_*r*_(*t*) ⊂ Ω_*e*_ ⊂ Ω. On the rest of the cortex not recruited in the seizure activity only noise is prescribed.

#### One source model

In the one homogeneous source model, the whole excitable patch is recruited at time *t*^0^,

$$\begin{array}{c} \Omega_{r}\left(t\right)=\{\begin{array}{cc} \emptyset & \text{if}\;t<t^{0},\\ \Omega_{e} & \text{if}\;t\geq t^{0}. \end{array} \end{array}$$ (1)

The excitable patch Ω_*e*_ is in each simulation selected randomly by choosing a center point on the cortex Ω which is placed closer than 15 mm from any of the electrode contacts. Then the patch size is chosen from its prescribed range (Table 2), and the patch is extended from the initial point until the desired size is reached. This is performed using a queue-based expansion: all neighboring vertices of the initial vertex are added to the queue, and then until the desired size is reached, first element from the queue is taken, added to the patch, and its neighbors not yet assigned to the patch are added at the end of the queue. Due to the possible inhomogeneous triangulation density, the created patches might not be circular even on a flat surface.

**Table 2:** Ranges and values of model parameters. Model abbreviations: OS—One Source, TS—Two Sources, SS—Spreading seizure. ^†^ For noise-free / noisy simulations.

Parameter	Model	Value
Size of the excitable patch |Ω_*s*_| [mm^2^]	OS, TS, SS	[400, 2500]
Frequency *f* [Hz]	OS, TS, SS	[4, 13]
Onset duration *δ* [s]	OS, TS	[1, 30]
Delay of the onset of the second patch Δ [s]	TS	[0, 10]
Spread velocity [mm s^−1^]	SS	[0.5, 4.0]
Wave velocity [mm s^−1^]	SS	[100, 500]
Scaling coefficient *q*^†^	OS	8.16 / 7.87
Scaling coefficient *q*^†^	TS	9.96 / 9.85
Scaling coefficient *q*^†^	SS	16.28 / 15.34

The source activity at point ***x*** on the cortical surface and time *t* is given by

$$\begin{array}{c} s\left(x,t\right)=\{\begin{array}{cc} q\;\min(1,\frac{t-t^{0}}{\delta })(y_{s}\left(t-t^{0}\right)+\alpha \nu \left(x,t\right)) & \text{if}\;x\in \Omega_{r}\left(t\right),\\ \eta (x,t) & \text{if}\;x\notin \Omega_{r}\left(t\right), \end{array} \end{array}$$ (2)

where

$$\begin{array}{c} y_{s}\left(t\right)=\{\begin{array}{cc} a_{s}\left(-2tf+1\right) & \text{if}\;tf-\lfloor tf\rfloor <\frac{1}{2},\\ a_{s}\left(2tf-3\right) & \text{elsewhere}, \end{array} \end{array}$$ (3)

is a triangle wave with frequency *f*, with power normalization constant $a_{s}=\sqrt{3}$. Next, *η*(***x***, *t*) and *ν*(***x***, *t*) is the background and seizure noise, described below, with the latter present only for the noisy simulations, i.e. *α* = 0 for noise-free and *α* = 1 for noisy simulations. Finally, *δ* is the duration of the onset pattern and *q* is a scaling coefficient (Table 2). We chose to use the triangle wave at the source to best approximate the typical waveform of theta-alpha activity, in literature sometimes described as “sharp” [6, 10].

#### Two sources model

In the two homogeneous sources model, there are two excitable patches which are recruited with delay Δ, so that $t_{2}^{0}=t_{1}^{0}+\Delta$ and

$$\begin{array}{c} \Omega_{r_{i}}\left(t\right)=\{\begin{array}{cc} \emptyset & \text{if}\;t<t_{i}^{0}\\ \Omega_{e_{i}} & \text{if}\;t\geq t_{i}^{0} \end{array}\;i=1,2. \end{array}$$ (4)

The excitable patches $\Omega_{e_{1}}$ and $\Omega_{e_{2}}$ are selected in a similar way as in the one source model. First, the patch centers are randomly chosen so that they lie closer than 15 mm from any of the electrode contacts and also closer to each other than 10 mm. Then the patch size is chosen, and each of the patches is expanded to half of its value. The source activity is given by

$$\begin{array}{c} s\left(x,t\right)=\{\begin{array}{cc} q\;\min(1,\frac{t-t_{1}^{0}}{\delta_{1}})(y_{s}\left(t-t_{1}^{0}\right)+\alpha \nu \left(x,t\right)) & \text{if}\;x\in \Omega_{r_{1}}\left(t\right),\\ q\;\min(1,\frac{t-t_{2}^{0}}{\delta_{2}})(y_{s}\left(t-t_{2}^{0}\right)+\alpha \nu \left(x,t\right)) & \text{if}\;x\in \Omega_{r_{2}}\left(t\right),\\ \eta (x,t) & \text{if}\;x\notin (\Omega_{r_{1}}\left(t\right)\cup \Omega_{r_{2}}\left(t\right)), \end{array} \end{array}$$ (5)

with the waveform *y*_*s*_ given by Eq 3, background and seizure noise *η*(***x***, *t*) and *ν*(***x***, *t*) described below, and onset duration *δ*_1_ and *δ*_2_ and scaling coefficient *q* as in Table 2.

#### Spreading seizure model

With the spreading seizure model, the excitable patch is recruited sequentially,

$$\begin{array}{c} \Omega_{r}\left(t\right)=\{\begin{array}{cc} \emptyset & \text{if}\;t<t^{0},\\ \left\{x\;|\;x\in \Omega_{s},\;d\left(x,x_{0}\right)<\left(t-t^{0}\right)\;u_{\text{spread}}\right\} & \text{if}\;t\geq t^{0}, \end{array} \end{array}$$ (6)

where *d*(***x***, ***x***_0_) is the geodesic distance of the point ***x*** from the seizure origin ***x***_0_ and *u*_spread_ is the velocity of seizure spread. The excitable patch is created the same way as in the one source model, and the seizure origin ***x***_0_ is selected randomly from all points on the excitable patch. The cortical activity is prescribed as

$$\begin{array}{c} s\left(x,t\right)=\{\begin{array}{cc} q(y_{p}\left(t-t^{0}-d\left(x,x_{0}\right)/u_{\text{wave}}\right)+\alpha \nu \left(x,t\right)) & \text{if}\;x\in \Omega_{r}\\ \eta (x,t) & \text{if}\;x\notin \Omega_{r} \end{array} \end{array}$$ (7)

where

$$\begin{array}{c} y_{p}\left(t\right)=\{\begin{array}{cc} a_{p} & \text{if}\;tf-\lfloor tf\rfloor <\tau f\\ 0 & \text{elsewhere.} \end{array} \end{array}$$ (8)

is a pulse wave with duty cycle *τ* = 0.25 and frequency *f*, $a_{p}=\sqrt{16/3}$ is the power normalization coefficient, the parameter *u*_wave_ is the velocity of the fast waves traveling across the recruited part of the excitable patch, *q* is the scaling coefficient, and *η*(***x***, *t*) and *ν*(***x***, *t*) is the background and seizure noise. The pulse waveform was chosen mainly for the simplicity, and other waveforms could have been used to model the traveling wave instead. However, since the subsequent analysis of the signals and the TAA detection is to a great extent oblivious to the specifics of the waveforms, we consider it unlikely that a different choice would lead to qualitatively different results.

#### Noise

The noise in the simulations consist of the background noise *η*(***x***, *t*) present in the regions not recruited into seizure activity, and (in case of noisy simulations) the seizure noise *ν*(***x***, *t*). Both the background and seizure noise are modeled as pink (i.e. 1/*f*) noise with power equal to one. The seizure noise is modeled as spatially correlated with kernel *k*(***x***, *x*′) = exp(−*d*(***x***, ***x***′)/*l*) with *l* = 10 mm, *d*(***x***, ***x***′) is the geodesic distance of two points on the cortical surface. For reasons of computational efficiency, the background noise of the whole cortex is not modeled as spatially correlated, instead, the cortex is divided into patches of average area 100 mm^2^, and the same noise time series is used for all points in one patch. The division is performed by randomly selecting appropriate number of seed vertices and then expanding all patches until the whole cortex is covered.

The scaling coefficient *q* was set experimentally so that the normalized log power of the simulated SEEG would have comparable maximal values as the normalized log power of the recorded signals. Specifically, for each model and for both noisy and noise-free simulations, sixty simulations with randomly chosen parameters were performed with fixed $\hat{q}=10$. Then the 80th percentile of log power normalized to preictal levels was calculated for all channels (across time), and 95 percentile *p*_sim_ was again calculated across all channels and simulated seizures. The same value *p*_rec_ was calculated for the recorded seizures, and the scaling coefficient was updated, $q=10^{\frac{p_{\text{rec}}-p_{\text{sim}}}{2}}\hat{q}$.

#### SEEG projection

In the human cortex, the most numerous neuron type is the pyramidal cell. Due to their geometrical structure with long dendrites oriented perpendicularly to the cortical surface, they might be well represented as electrical dipoles [25]. Following this idea, we assume that each point on the cortical surface acts as electrical dipole. In the spatially continuous formulation the local field potential measured by the electrode contact at point ***x***_s_ generated by the source activity *s*(***x***, *t*) on the surface Ω is given by

$$\begin{array}{ccc} \phi (x_{s},t) & = & \int_{\Omega }\frac{n\cdot \left(x_{s}-x\right)/\left|x_{s}-x\right|}{\left(\right|x_{s}-x{\left|+\epsilon \right)}^{2}}s\left(x,t\right)\;dx, \end{array}$$ (9)

where ***n*** is the outward oriented normal of the surface, and the constant *ϵ* = 1 mm is added to prevent the singularities at the surface. In the discretized version using the calculated solution on a triangulated surface the formula is instead

$$\begin{array}{ccc} \phi (x_{s},t) & \approx & \sum_{v\in V}A_{v}\frac{n_{v}\cdot \left(x_{s}-x_{v}\right)/\left|x_{s}-x\right|}{{\left(|x_{s}-x_{v}|+\epsilon \right)}^{2}}s_{v}\left(t\right), \end{array}$$ (10)

where *V* is the set of all vertices on the triangulated surface, *A*_v_ is the area associated with a vertex (calculated as one third of the sum of areas of neighboring triangles), ***n***_v_ is the outwards oriented normal of a vertex (calculated as a weighted average of the normals of neighboring triangles), ***x***_v_ is the position of the vertex, and *s*_v_(*t*) is the activity at the vertex prescribed by one of the above models (Eqs (2), (5) or (7)).

### TAA pattern detection

The occurrences of the TAA pattern in all channels of the recorded (or simulated) electrographic signals are detected in two steps. In the first step, we look for the temporal interval where the power in the *θ* − *α* band grows from the preictal baseline to full seizure activity. In the second step, we check that this tentative interval satisfies further conditions on the TAA pattern, namely that the oscillatory activity is dominated by single frequency oscillations in the *θ* − *α* range and the growth is close to linear.

The first step is implemented as follows. The power in the *θ* − *α* band (4 to 13 Hz) of the SEEG signals in monopolar representation is calculated using the multitaper method (time bandwidth = 2.0, number of cycles = 8). The *θ* − *α* log-power *LP*_*θ* − *α*_ is then normalized to preictal baseline (calculated from sixty seconds preceding the clinically marked seizure onset) so that 〈*LP*_*θ* − *α*_〉_*preictal*_ = 0. The channel is marked as *seizing* if the 90-th percentile of the power *P*_90_ is at least *k*_s_-times larger than the baseline with *k*_s_ = 30. If it is not seizing, than it is also marked as not TAA. Otherwise, the limits of the tentative interval of the TAA pattern [*t*^*o*^, *t*^*t*^] are set as *t*^*t*^ = min{*t* | *LP*_*θ*−*α*_(*t*) < *k*_2_ *P*_90_} and *t*^*o*^ = max{*t* | *t* < *t*^*t*^, *LP*_*θ*−*α*_(*t*) > *k*_1_ *P*_90_}, where the lower threshold coefficient is set to *k*_1_ = 0.15 and upper threshold coefficient to *k*_2_ = 0.85. This procedure implies that this tentative TAA interval can be preceded by any activity as long as its *θ* − *α* log-power does not cross the upper threshold; the TAA patterns that we detect can thus appear after abnormal electrographic activity of low power or outside of the *θ* − *α* band.

Next, we check that the signal in the tentative interval satisfies our criteria on TAA patterns. First, we apply the linear regression on the log-power over time, and check that the coefficient of determination is sufficiently high, *R*^2^ > 0.75. Second, we calculate the power spectral density of the signal in the tentatively determined interval in the range 1 to 100 Hz, we flatten the spectrum by multiplying it by the frequencies, and we normalize it so that the maximum is equal to one. We then detect the peaks in the spectrum (minimum peak height 0.25, peak distance 2 Hz). The pattern is confirmed to be the TAA pattern if the frequency of the largest peak *f*_0_ lies in the *θ* − *α* range (i.e. 4 to 13 Hz), and if all other peaks *f*_i_ are harmonics of *f*_0_ (with tolerance 0.15*f*_0_, i.e |*f*_i_ − *kf*_0_| < 0.15*f*_0_ for some *k* = 1, 2, …).

When the TAA pattern is detected on four or more neighboring contacts of a single electrode, five features are computed for the group. Two are obtained from linear regression of the TAA onset times *t*^*o*^ w.r.t. the position on the electrode: *slope* and the coefficient of determination *R*^2^. Next is the average *duration* of the TAA pattern, i.e. ${\langle t_{i}^{t}-t_{i}^{o}\rangle }_{i}$, where *i* indexes the contacts in the group. The last two features are determined by performing a principal component analysis of the recorded (or simulated) signals in the interval $\left[\min_{i}t_{i}^{o},\max_{i}t_{i}^{t}\right]$, and calculating the variance explained by the first one (*PCA VE1*) and first two (*PCA VE2*) components.

### Measures of goodness-of-fit

We use three measures to quantify the fit between the features of the TAA patterns detected in the recordings and in the simulated data sets. In the following paragraphs, we assume that from the recordings we have *n* *d*-dimensional samples $X_{p}={\left\{x_{p,i}\right\}}_{i=1}^{n}$ from the probability distribution *P* with unknown probability density function *p*, and that we have *m* model-generated samples $X_{q}={\left\{x_{q,i}\right\}}_{i=1}^{m}$ from the distribution *Q* with probability density function *q*.

#### Log-likelihood

The log-likelihood measures how likely are the samples *X*_p_ under the hypothesis represented by the probability distribution *Q*,

$$\begin{array}{c} \log q\left(X_{p}\right)=\sum_{i=1}^{n}\log q\left(x_{p,i}\right). \end{array}$$ (11)

The probability density function *q* is however not known, and we approximate it with its *k*-nearest-neighbor estimation,

$$\begin{array}{c} LL_{k}=\sum_{i=1}^{n}\log\hat{q}\left(x_{p,i}\right)=\sum_{i=1}^{n}\log\frac{k\;\Gamma (d/2+1)}{m\;\pi^{d/2}s_{k}\left(x_{p,i},x_{q}\right)} \end{array}$$ (12)

where Γ is the gamma function and *s*_k_ is the distance from *x*_*p*,*i*_ to its *k*-nearest-neighbor among *X*_q_.

#### Bhattacharyya distance

The Bhattacharyya distance [53] measures the overlap of two probability distributions. It relies on the binning of the samples. Given the probabilities *p*_*j*_ and *q*_*j*_ in *j* = 1 … *n*_*b*_ bins, the distance is defined as

$$\begin{array}{c} D_{B}\left(P,Q\right)=-\log\left(BC\left(P,Q\right)\right), \end{array}$$ (13)

where $BC\left(P,Q\right)=\sum_{j=1}^{n_{b}}\sqrt{p_{j}q_{j}}$ is the Bhattacharyya coefficient.

#### Earth mover’s distance

Intuitively, Earth mover’s distance [54] corresponds to minimal amount of work needed to transport the mass from one distribution to another. Again, we need to bin the samples into bins to obtain the probabilities *p*_*j*_ and *q*_*j*_ in *j* = 1 … *n*_*b*_ bins. Then the Earth Mover’s distance is defined as

$$\begin{array}{c} D_{EMD}\left(P,Q\right)=\min_{f}\sum_{i=1}^{n_{b}}\sum_{j=1}^{n_{b}}d_{ij}f_{ij}, \end{array}$$ (14)

where *d*_ij_ is the Euclidean distance between the centers of *i*-th and *j*-th bin. The distances along each dimensions are normalized by the standard deviation of the feature values in the recording samples. The cost function is minimized over all possible flows $f={\left\{f_{ij}\right\}}_{i,j=1}^{n_{b}}$, subject to following constraints:

$$f_{ij}\geq 0\;i,j=1,\ldots ,n_{b},$$ (15)

$$\sum_{j=1}^{n_{b}}f_{ij}=q_{j}\;i=1,\ldots ,n_{b},$$ (16)

$$\sum_{i=1}^{n_{b}}f_{ij}=p_{j}\;j=1,\ldots ,n_{b}.$$ (17)

The constraint (15) guarantees that only positive amount of mass is transported, while (16) and (17) limit the amount of mass transported from and to a single bin to the amount given by the distributions. The optimization is implemented using the OR-Tools package [55].

## Supporting information

S1 Fig Study of the mesh dependence. (A-D) Sixty simulations of spreading seizure in the noise-free variant of the model were performed on the standard triangulation (described in the main text) and on refined triangulation of the cortex, obtained by splitting every existing triangle into four. The simulation parameters were chosen randomly as in the main text, but were kept between the simulations on standard and fine triangulations, so that the results are directly comparable. The background noise was however different between the simulations on standard and fine triangulation. (A) The activity of the simulated SEEG signals were classified into non-seizing (NSZ), seizing but not TAA (SZ), and TAA (see Methods). (B) For the signals classified as TAA in both standard and fine simulations, the determined time of TAA onset is compared. Perfect fit would lie on the diagonal marked by black dashed line. (C) Same as B but for the determined duration of TAA pattern. (D) Histogram of the differences of the TAA onset times and durations from panels B and C. The range is clipped for visualization, amount of clipped values is shown in the inset text. (E-F) To assess the influence of the background noise, second set of simulations on the standard triangulation was performed. The parameters of the simulations were again kept the same as in the first set, only with the background noise changed. Panels are equivalent to panels A-D, showing the fit between the two sets of simulations on standard triangulations. Comparison between the first and second row of the figure indicates that the differences between the simulations on standard and fine triangulations are caused mainly by the stochastic background noise, since they are present also for the simulations on the same triangulations. The level of mesh refinement thus does not introduce differences of higher order of magnitude.
(PDF)

S2 Fig Properties of non-group TAA instances. A TAA instance is classified as belonging to group if it is one of at least four TAA instances on neighboring contacts of the same electrode. (A) Number of detected TAA instances on the same electrode for each non-group TAA instance. For each detected non-group TAA, we counted the number of TAA instances on the same electrode. Instances that occur in isolation represent 29% of non-group TAA instances and 21% of all TAA instances. A non-group TAA instance can occur on one electrode with more than three other TAA instances if they are non-contiguous. (B) Frequency, duration, and delay from the seizure onset for the detected non-group TAA instances (*n* = 448) and the group TAA instances (*n* = 160). Statistical analysis (Mann-Whitney U-test) does not indicate a difference in frequencies (*U* = 34639.5, *p* = 0.265), durations (*U* = 33969.0, *p* = 0.163), or delays (*U* = 33732.5, *p* = 0.135).
(PDF)

S3 Fig Distance of the electrode contacts to the suspected epileptogenic zone. Plotted are the histograms and kernel density estimates of distances of contacts with detected seizure activity, with non-group TAA instances, and with group TAA instances. For the purpose of this figure, we considered the epileptogenic zone to be located at the contact pair with the highest calculated Epileptogenicity index (EI) [27]. Note however that the EI was calculated only for some seizures (25.4%); for others we used the EI from other seizure in the same subject (54.4%), and yet other seizures where EI was not available for the subject were excluded from this analysis (20.1%). Results indicate that the contacts with the group and non-group TAA instance follow the same distribution of distances as all seizing contacts.
(PDF)

S4 Fig Similarity and variability of the detected TAA instances. We applied the k-means clustering on the normalized features of the detected TAA instances. We applied the k-means clustering on the normalized features of the detected TAA instances. (A) Optimal numbers of clusters, assessed by the second derivative of sum of squared deviations (SSD, lower is better), silhouette score (higher is better), and Calinski-Harabasz score (higher is better). Taking the three criteria into account, we identify five clusters as optimal. (B) The clusters can be roughly described as: TAA instances with large duration (cluster 2), instances with low variance explained by first two PCA components, either with small slope (cluster 1) or large slope (cluster 4), and instances with high variance explained, either with small slope and low *R*^2^ (cluster 3) or varying slope and high *R*^2^ (cluster 5). The difference between the latter two clusters might not be meaningful, as the coefficient of determination *R*^2^ does not convey useful information when the slope is small. (C) Detected TAA instances in individual subjects. The numbers refer to the S1 Table, each circle represents a detected TAA instance with coloring corresponding to the clusters in panel B, and each row represents one seizure. None of the clusters is specific to a single subject, and no subject is thus clear outlier from the rest of the data set.
(PDF)

S5 Fig Stability of the main results with respect to the parameters of the TAA detection procedure. Figure shows the confidence intervals of the differences of the log-likelihoods to those of noisy spreading seizure model. The log-likelihoods are estimated by *k*-nearest-neighbor approximation with *k* = 10. The charts thus correspond to the lower left panel of Fig 5B in the main text. (A-C) Results when systematically varying three parameters of the TAA detection procedure (seizure threshold *k*_s_, A; lower threshold *k*_1_, B; and upper threshold *k*_2_, C), while keeping the other two at its default values. (D-E) Results for the default detection procedure, but with modified range of the model parameters frequency and patch size. The default parameter values are given in Table 2 in the main text. Although quantitative differences exists, qualitatively the results for most parameter values or ranges agree, with the exception of the patch size. For small patch sizes both the two source model and one source model are also plausible. That can be explained by considering that the with reduction of the size the activity on the patch in the spreading seizure model becomes more homogeneous, thus less distinguishable from the homogeneous source models. Abbreviation of the model names: OS—One homogeneous source, TS—Two homogeneous sources, SS—Spreading seizure.
(PDF)

S6 Fig Properties of non-group TAA instances. Effects of the parameters in the spreading seizure model in the noisy variant, visualizing the relations on Fig 6A in the main text. Each panel shows the relation between one parameter and one feature. Solid line and points represent the mean of the features, the shaded area is the 10-90 percentile range. The last row shows the histogram of the parameters among the detected TAAs.
(PDF)

S1 Table Patient table. (PDF)
