# Supplementary material for: Evidence for spreading seizure as a cause of theta-alpha activity electrographic pattern in stereo-EEG seizure recordings
Source: PLoS Comput Biol. 2021 Feb 26;17(2):e1008731. doi: 10.1371/journal.pcbi.1008731 (PMC7946361; doi:10.1371/journal.pcbi.1008731)
Supplement: S1 Fig — (A-D) Sixty simulations of spreading seizure in the noise-free variant of the model were performed on the standard triangulation (described in the main text) and on refined triangulation of the cortex, obtained by splitting every existing triangle into four. The simulation parameters were chosen randomly as in the main text, but were kept between the simulations on standard and fine triangulations, so that the results are directly comparable. The background noise was however different between the simulations on standard and fine triangulation. (A) The activity of the simulated SEEG signals were classified into non-seizing (NSZ), seizing but not TAA (SZ), and TAA (see Methods). (B) For the signals classified as TAA in both standard and fine simulations, the determined time of TAA onset is compared. Perfect fit would lie on the diagonal marked by black dashed line. (C) Same as B but for the determined duration of TAA pattern. (D) Histogram of the differences of the TAA onset times and durations from panels B and C. The range is clipped for visualization, amount of clipped values is shown in the inset text. (E-F) To assess the influence of the background noise, second set of simulations on the standard triangulation was performed. The parameters of the simulations were again kept the same as in the first set, only with the background noise changed. Panels are equivalent to panels A-D, showing the fit between the two sets of simulations on standard triangulations. Comparison between the first and second row of the figure indicates that the differences between the simulations on standard and fine triangulations are caused mainly by the stochastic background noise, since they are present also for the simulations on the same triangulations. The level of mesh refinement thus does not introduce differences of higher order of magnitude. (PDF) [file pcbi.1008731.s001.pdf]

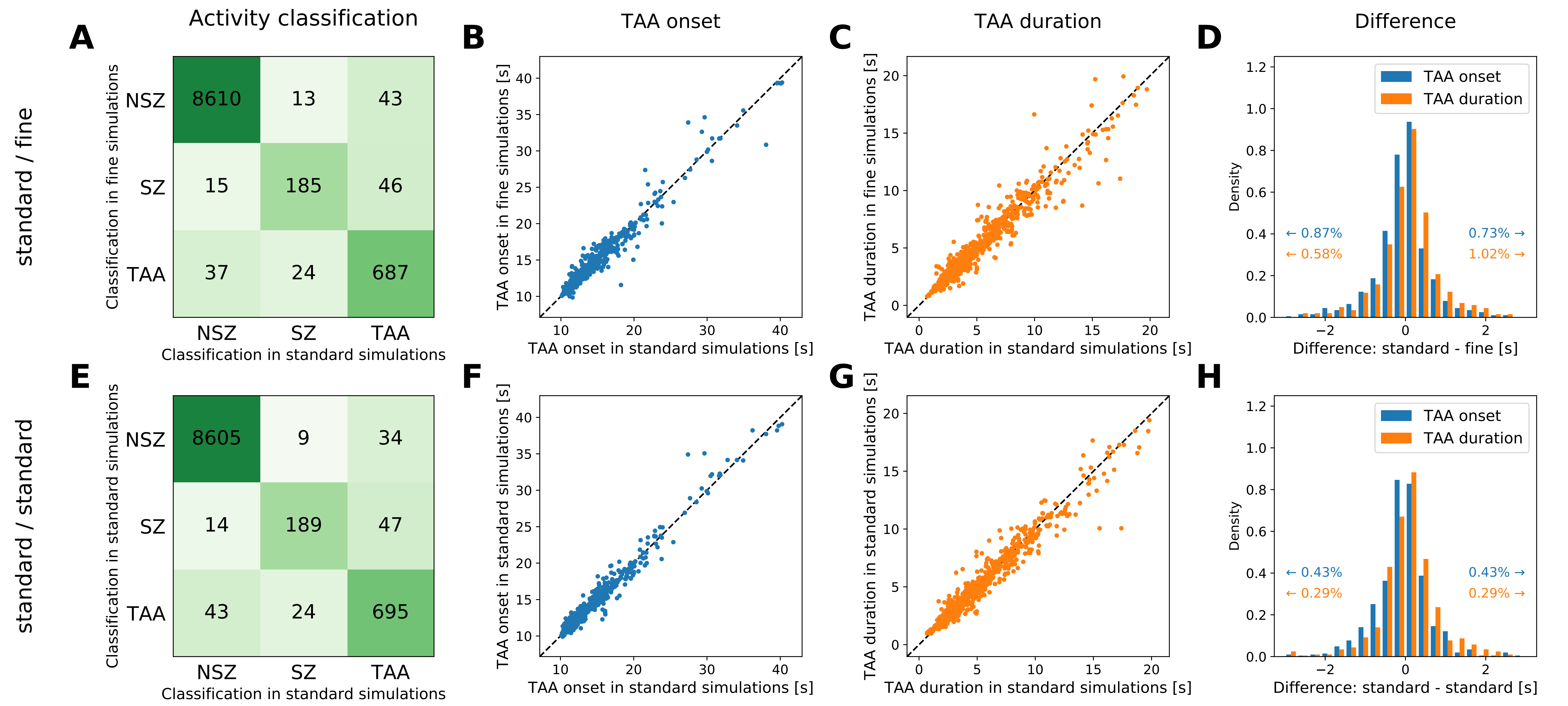
Activity classification
TAA onset
TAA duration
Difference
A
B
C
D
E
F
G
H
standard / fine
standard / standard
Classification in fine simulations
Classification in standard simulations
NSZ
SZ
TAA
8610
13
43
15
185
46
37
24
687
8605
9
34
14
189
47
43
24
695
TAA onset in fine simulations [s]
TAA onset in standard simulations [s]
TAA duration in fine simulations [s]
TAA duration in standard simulations [s]
Density
TAA onset
TAA duration
← 0.87%
0.73% →
← 0.58%
1.02% →
← 0.43%
0.43% →
← 0.29%
0.29% →
Difference: standard - fine [s]
Difference: standard - standard [s]
