# Supplementary figures and images for: Evidence for spreading seizure as a cause of theta-alpha activity electrographic pattern in stereo-EEG seizure recordings
Source: PLoS Comput Biol. 2021 Feb 26;17(2):e1008731. doi: 10.1371/journal.pcbi.1008731 (PMC7946361; doi:10.1371/journal.pcbi.1008731)

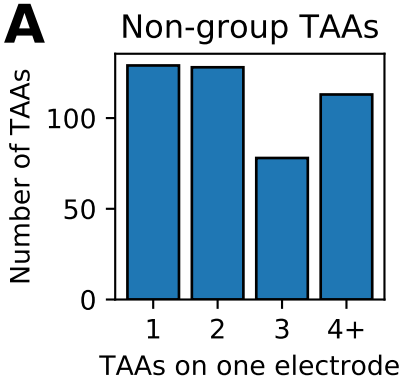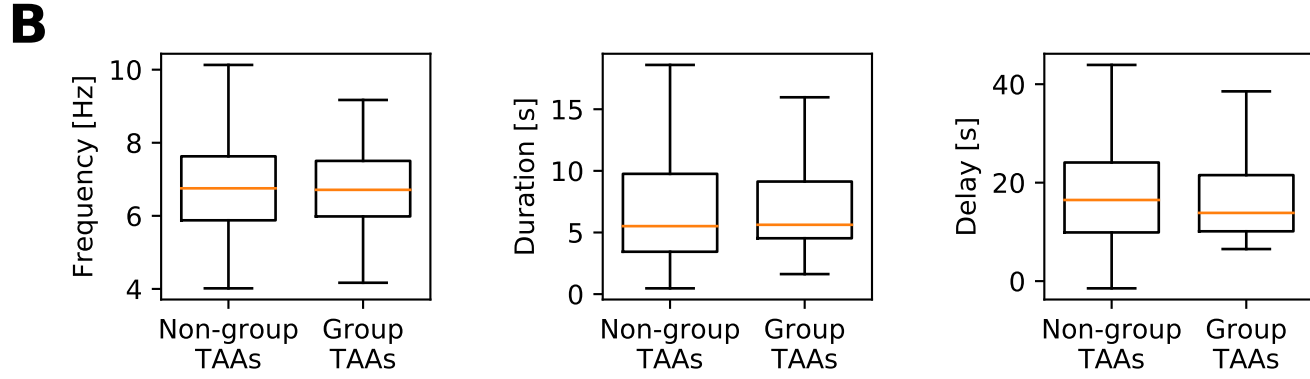

Supplement: S2 Fig — A TAA instance is classified as belonging to group if it is one of at least four TAA instances on neighboring contacts of the same electrode. (A) Number of detected TAA instances on the same electrode for each non-group TAA instance. For each detected non-group TAA, we counted the number of TAA instances on the same electrode. Instances that occur in isolation represent 29% of non-group TAA instances and 21% of all TAA instances. A non-group TAA instance can occur on one electrode with more than three other TAA instances if they are non-contiguous. (B) Frequency, duration, and delay from the seizure onset for the detected non-group TAA instances (n = 448) and the group TAA instances (n = 160). Statistical analysis (Mann-Whitney U-test) does not indicate a difference in frequencies (U = 34639.5, p = 0.265), durations (U = 33969.0, p = 0.163), or delays (U = 33732.5, p = 0.135). (PDF) [file pcbi.1008731.s002.pdf]

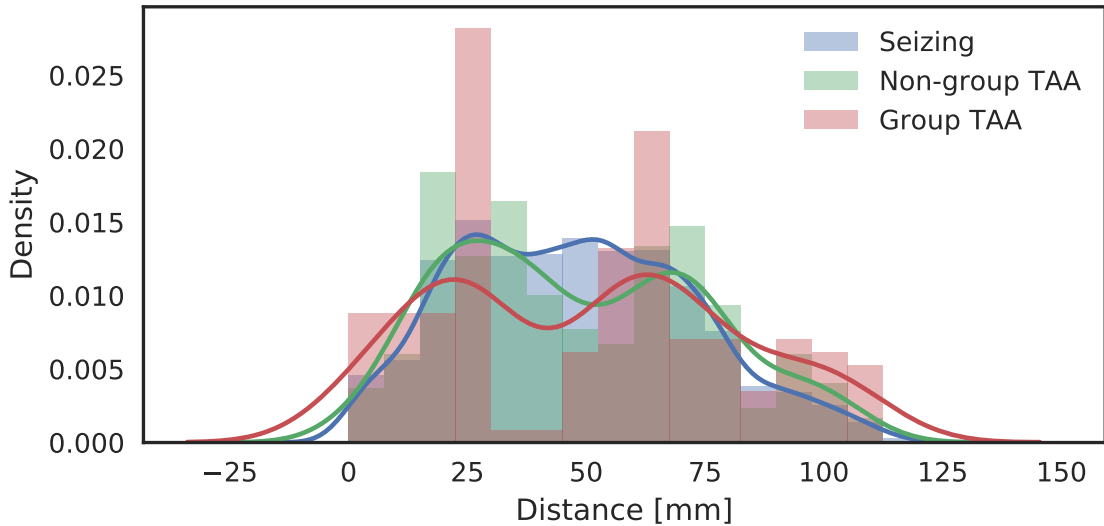

Supplement: S3 Fig — Plotted are the histograms and kernel density estimates of distances of contacts with detected seizure activity, with non-group TAA instances, and with group TAA instances. For the purpose of this figure, we considered the epileptogenic zone to be located at the contact pair with the highest calculated Epileptogenicity index (EI) [27]. Note however that the EI was calculated only for some seizures (25.4%); for others we used the EI from other seizure in the same subject (54.4%), and yet other seizures where EI was not available for the subject were excluded from this analysis (20.1%). Results indicate that the contacts with the group and non-group TAA instance follow the same distribution of distances as all seizing contacts. (PDF) [file pcbi.1008731.s003.pdf]

**A**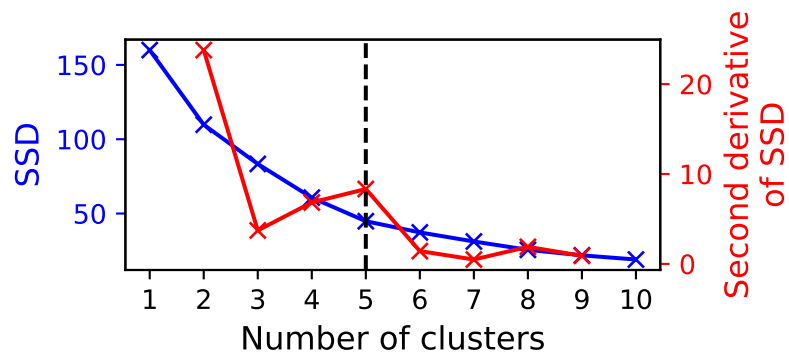

Silhouette score

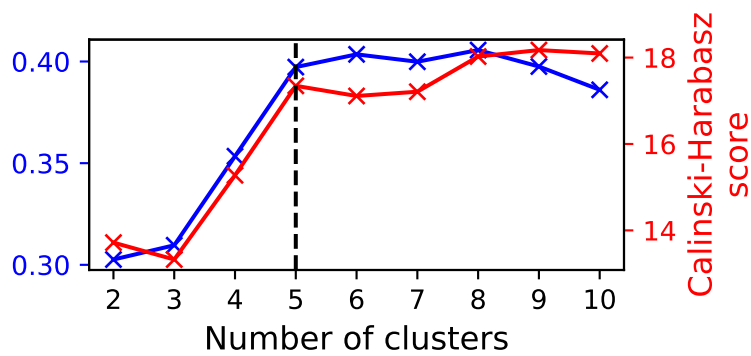**C**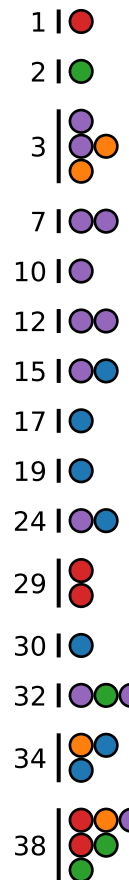**B**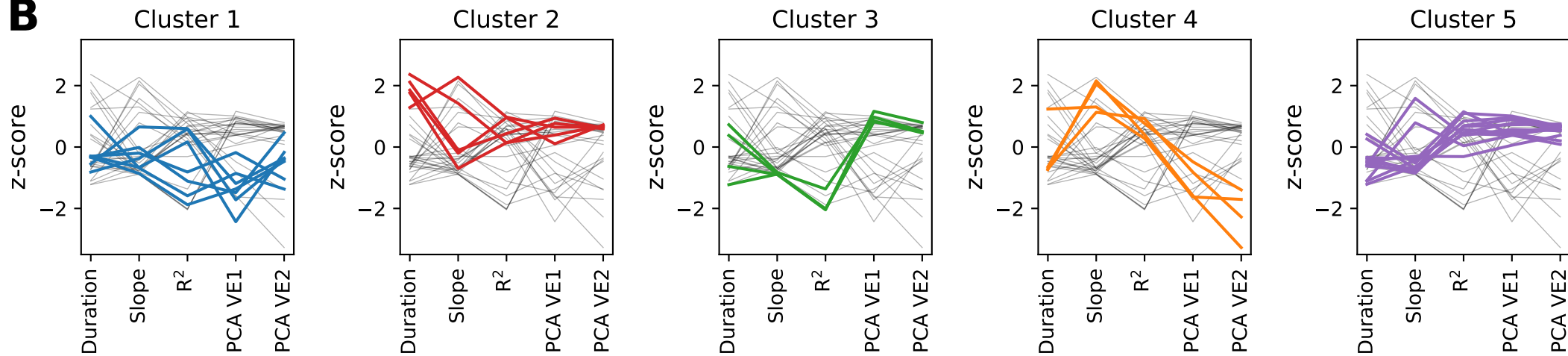

Supplement: S4 Fig — We applied the k-means clustering on the normalized features of the detected TAA instances. (A) Optimal numbers of clusters, assessed by the second derivative of sum of squared deviations (SSD, lower is better), silhouette score (higher is better), and Calinski-Harabasz score (higher is better). Taking the three criteria into account, we identify five clusters as optimal. (B) The clusters can be roughly described as: TAA instances with large duration (cluster 2), instances with low variance explained by first two PCA components, either with small slope (cluster 1) or large slope (cluster 4), and instances with high variance explained, either with small slope and low R2 (cluster 3) or varying slope and high R2 (cluster 5). The difference between the latter two clusters might not be meaningful, as the coefficient of determination R2 does not convey useful information when the slope is small. (C) Detected TAA instances in individual subjects. The numbers refer to the S1 Table, each circle represents a detected TAA instance with coloring corresponding to the clusters in panel B, and each row represents one seizure. None of the clusters is specific to a single subject, and no subject is thus clear outlier from the rest of the data set. (PDF) [file pcbi.1008731.s004.pdf]

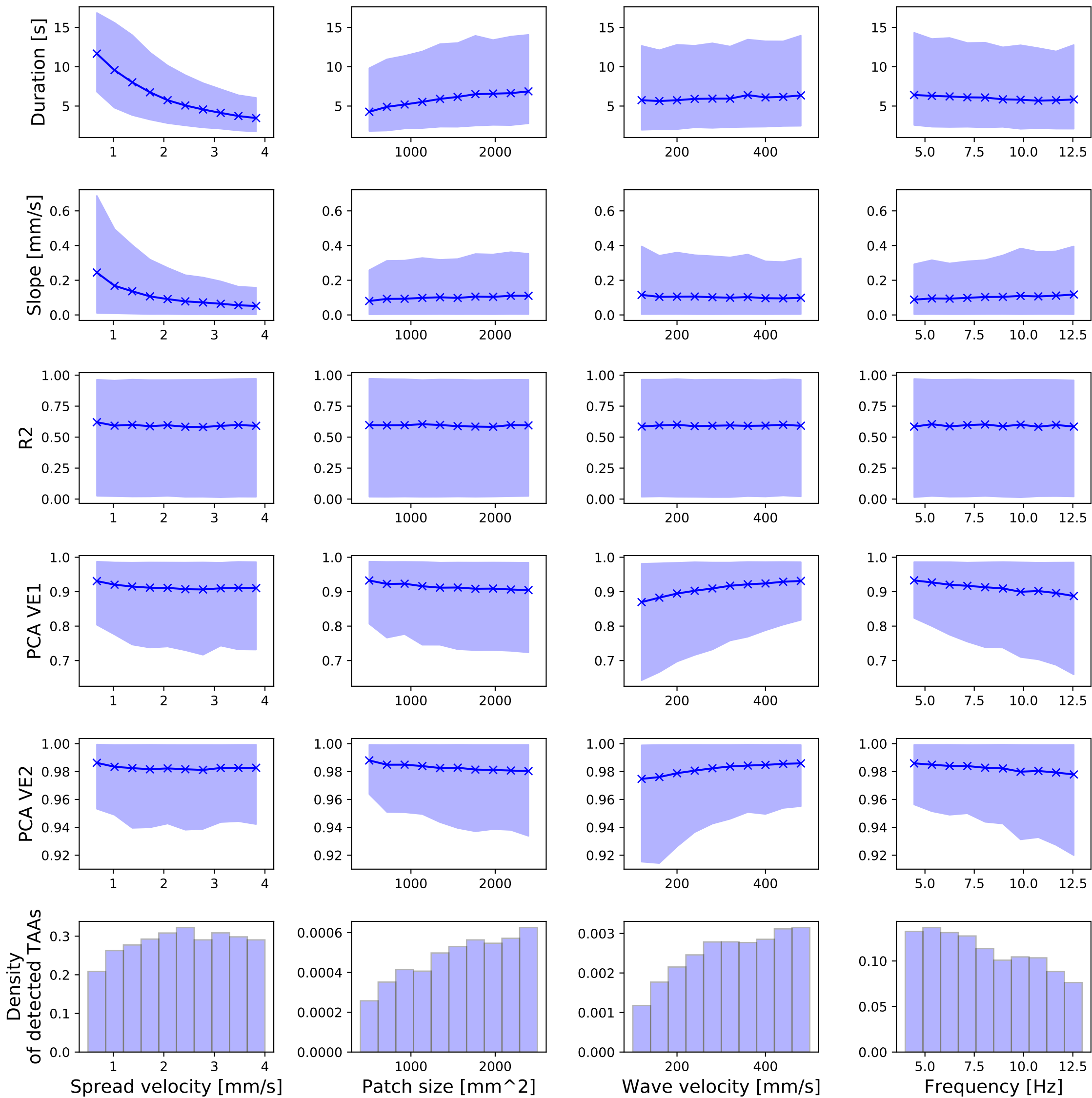

Supplement: S6 Fig — Effects of the parameters in the spreading seizure model in the noisy variant, visualizing the relations on Fig 6A in the main text. Each panel shows the relation between one parameter and one feature. Solid line and points represent the mean of the features, the shaded area is the 10-90 percentile range. The last row shows the histogram of the parameters among the detected TAAs. (PDF) [file pcbi.1008731.s006.pdf]
