# Supplementary material for: Evidence for spreading seizure as a cause of theta-alpha activity electrographic pattern in stereo-EEG seizure recordings
Source: PLoS Comput Biol. 2021 Feb 26;17(2):e1008731. doi: 10.1371/journal.pcbi.1008731 (PMC7946361; doi:10.1371/journal.pcbi.1008731)
Supplement: S5 Fig — Figure shows the confidence intervals of the differences of the log-likelihoods to those of noisy spreading seizure model. The log-likelihoods are estimated by k-nearest-neighbor approximation with k = 10. The charts thus correspond to the lower left panel of Fig 5B in the main text. (A-C) Results when systematically varying three parameters of the TAA detection procedure (seizure threshold ks, A; lower threshold k1, B; and upper threshold k2, C), while keeping the other two at its default values. (D-E) Results for the default detection procedure, but with modified range of the model parameters frequency and patch size. The default parameter values are given in Table 2 in the main text. Although quantitative differences exists, qualitatively the results for most parameter values or ranges agree, with the exception of the patch size. For small patch sizes both the two source model and one source model are also plausible. That can be explained by considering that the with reduction of the size the activity on the patch in the spreading seizure model becomes more homogeneous, thus less distinguishable from the homogeneous source models. Abbreviation of the model names: OS—One homogeneous source, TS—Two homogeneous sources, SS—Spreading seizure. (PDF) [file pcbi.1008731.s005.pdf]

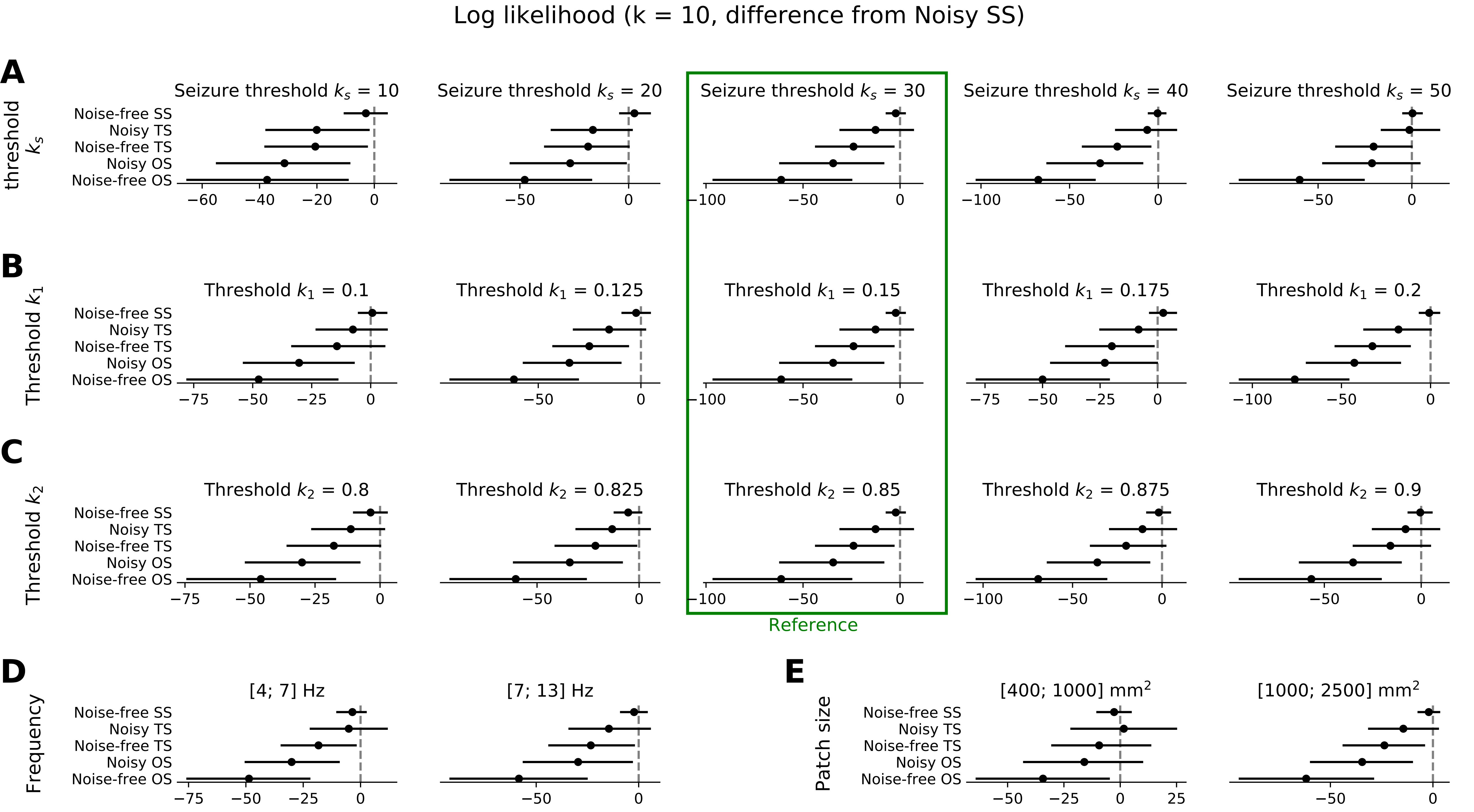

Log likelihood (k = 10, difference from Noisy SS)
A
threshold k_s
Seizure threshold k_s = 10
Seizure threshold k_s = 20
Seizure threshold k_s = 30
Seizure threshold k_s = 40
Seizure threshold k_s = 50
Noise-free SS
Noisy TS
Noise-free TS
Noisy OS
Noise-free OS
B
Threshold k_1
Threshold k_1 = 0.1
Threshold k_1 = 0.125
Threshold k_1 = 0.15
Threshold k_1 = 0.175
Threshold k_1 = 0.2
C
Threshold k_2
Threshold k_2 = 0.8
Threshold k_2 = 0.825
Threshold k_2 = 0.85
Threshold k_2 = 0.875
Threshold k_2 = 0.9
Reference
D
Frequency
[4; 7] Hz
[7; 13] Hz
E
Patch size
[400; 1000] mm^2
[1000; 2500] mm^2
