# Supplementary material for: Evidence for spreading seizure as a cause of theta-alpha activity electrographic pattern in stereo-EEG seizure recordings
Source: PLoS Comput Biol. 2021 Feb 26;17(2):e1008731. doi: 10.1371/journal.pcbi.1008731 (PMC7946361; doi:10.1371/journal.pcbi.1008731)
Supplement: S1 Table — (PDF) [file pcbi.1008731.s007.pdf]

Supporting information: Evidence for spreading seizure as a cause of theta-alpha activity electrographic pattern in stereo-EEG seizure recordings

Viktor Sip[1] Julia Scholly[2,3] Maxime Guye[2,4] Fabrice Bartolomei[1,3] Viktor Jirsa[1*]

1 Aix Marseille Univ, INSERM, INS, Inst Neurosci Syst, Marseille, France
2 Assistance Publique - Hôpitaux de Marseille, Hôpital de la Timone, CEMEREM, Pôle d'Imagerie Médicale, CHU, Marseille, France
3 Assistance Publique - Hôpitaux de Marseille, Hôpital de la Timone, Service de Neurophysiologie Clinique, CHU, Marseille, France
4 Aix Marseille Univ, CNRS, CRMBM, Marseille, France

* viktor.jirsa@univ-amu.fr

Table 1. Patient table. Abbreviations: AVM, arteriovenous malformation; DNET, dysembryoplastic neuroepithelial tumor; FCD, focal cortical dyplasia; HH, hypothalamic hamartoma; L, left; NA, not applicable; PMG, polymicrogyria; PNH, periventricular nodular heterotopia; R, right.

Patient	Gender	Age at epilepsy onset (y)	Epilepsy duration (y)	Epilepsy type	MRI	Histopathology	Side	Number of seizures	Detected TAA groups
1	F	31	3	Temporo-insular	Normal	Hippocampal sclerosis	R	4	1
2	F	19	10	Temporo-occipital	L temporo-occipital PNH	NA	L	9	1
3	M	23	13	Temporo-frontal	R temporo-occipital scar	FCD1a	R	3	4
4	F	23	3	Temporal	R temporal mesial ganglioglioma	Ganglioglioma	R	4	0
5	M	0.3	21	Postcentral - superior parietal	L postcentral-parietal gyration asymmetry	NA	L	3	0
6	M	45	14	Fronto-temporal	Normal	NA	L	4	0
7	M	55	5	Temporal	Normal	Slight gliosis	R¿L	2	2
8	F	38	8	Temporal	L amygdala enlargement	Slight gliosis	L	4	0
9	F	11	34	Bifocal: parietal mesial & temporo-basal	Unknown R parietal lesion	Rosenthal fibers; slight gliosis	R	5	0
10	F	27	18	Temporal	L hippocampal sclerosis	Hippocampal sclerosis	L	7	1
11	F	27	14	Frontal	L frontal scar (abcess)	Gliosis	L	3	0
12	F	19	9	Bilateral temporo-frontal	Bilateral hippocampal & amygdala T2-hypersignal	NA	R&L	2	2
13	M	2	17	Frontal	Normal	Slight gliosis	L	3	0
14	F	5	18	Premotor	Normal	FCD2b	L	6	0
15	M	8	33	Temporal	R temporal PMG & multiple PNH	NA	R	2	2
16	M	6	23	Temporo-operculo-fronto-parietal	R temporo-parieto-insular & L temporo-parietal necrosis	NA	R¿L	5	0
17	M	5	21	Temporal	L temporo-polar hypothrophy and hippocampal sclerosis	Hippocampal sclerosis; gliosis	L	4	1
18	M	2	22	Parieto-temporal	L Parieto-occipital necrosis (perinatal anoxy)	NA	L	2	0
19	M	29	15	Temporo-insular	Normal	NA	L¿R	5	1
20	F	17	10	Temporal	Normal	Hippocampal sclerosis	R	3	0
21	F	9	14	Occipital	Normal	FCD1c	L	4	0
22	F	7	23	Parietal	L parietal FCD	FCD2b	L	5	0
23	M	35	28	Temporal	Normal	Gliosis	L	2	0
24	M	14	15	Temporal	Normal	NA	R	4	2
25	M	7	35	Insular	Normal	NA	L	4	0
26	F	4	24	Occipital	PNH	NA	R	5	0
27	M	17	12	Frontal	R prefrontal gliotic scar (AVM)	Gliosis	R¿L	3	0
28	F	8	14	Temporo-frontal	Anterior temporal necrosis	Gliosis	R	3	0
29	F	21	9	Bilateral temporal	Bilateral posterior PNH	NA	R¿L	10	2
30	M	11	45	Temporo-frontal	R Frontal FCD	FCD 2	R	2	1

Table 1. Patient table (continued).

Patient	Gender	Age at epilepsy onset (y)	Epilepsy duration (y)	Epilepsy type	MRI	Histopathology	Side	Number of seizures	Detected TAA groups
31	F	20	18	Occipital	Normal	NA	R	4	0
32	F	15	21	Bilateral temporal with HH	L HH	NA	R&L	5	3
33	F	18	5	Temporo-parieto-opercular	Normal	Hippocampal sclerosis	R	10	0
34	F	33	8	Temporal	Multiple R temporo-parietal PNH & temporal PMG	NA	R	2	3
35	M	4	27	Bilateral occipito-temporal	R occipital mesial FCD	NA	R&L	4	0
36	F	8	13	Temporo-insular	R temporal anterior resection cavity	Gliosis	R	4	0
37	M	28	5	Temporal mesial	R temporo-polar & amygdala FCD, L post-chiasmal pilocytic astrocyrtoma	FCD 2b	R	4	0
38	M	11	22	Bilateral temporal	Normal	NA	L&R	6	6
39	M	40	4	Temporo-frontal	R fronto-temporal necrosis (gunshot injury)	Gliosis	R	3	0
40	F	16	19	Temporal mesial	Hippocampal sclerosis	Hippocampal sclerosis	L	2	0
41	M	0.7	26	Bilateral, temporal predominant	R perisylvian necrosis (perinatal stroke)	NA	R¿L	4	0
42	F	9	19	Temporal mesial	Bilateral hippocampal sclerosis	NA	L	6	0
43	F	7	16	Premotor	R precentral FCD	NA	R	3	0
44	M	0.5	39	Multifocal: parieto-operculo-premotor; temporal mesial	L hippocampal & amygdala T2 hypersignal	NA	L	2	0
45	F	24	17	Temporal mesial	Normal	NA	L	4	0
46	M	1.5	31	Insulo-parieto-premotor	Normal	NA	R	7	0
47	M	16	13	Bilateral frontal	Normal	NA	R&L	5	0
48	F	15	7	Premotor	R parietal DNET	NA	R	3	0
49	F	1	21	Motor-opercular	R fronto-opercular resection cavity	NA	R	1	0
50	M	14	21	Motor-premotor	L insulo-opercular necrosis (stroke)	NA	L	3	0
